# Bioactive Film‐Guided Soft–Hard Interface Design Technology for Multi‐Tissue Integrative Regeneration

**DOI:** 10.1002/advs.202105945

**Published:** 2022-03-23

**Authors:** Yamin Li, Can Chen, Jia Jiang, Shengyang Liu, Zeren Zhang, Lan Xiao, Ruixian Lian, Lili Sun, Wei Luo, Michael Tim‐yun Ong, Wayne Yuk‐wai Lee, Yunsu Chen, Yuan Yuan, Jinzhong Zhao, Changsheng Liu, Yulin Li

**Affiliations:** ^1^ Engineering Research Centre for Biomedical Materials of Ministry of Education The Key Laboratory for Ultrafine Materials of Ministry of Education School of Material Science and Engineering Frontiers Science Center for Materiobiology and Dynamic Chemistry East China University of Science and Technology Shanghai 200237 China; ^2^ Shanghai Jiao Tong University Affiliated Sixth People's Hospital Shanghai 200233 China; ^3^ Centre for Biomedical Technologies Queensland University of Technology The Australia‐China Centre for Tissue Engineering and Regenerative Medicine (ACCTERM) 60 Musk Avenue, Kelvin Grove Brisbane QLD 4059 Australia; ^4^ Department of Orthopaedics and Traumatology Faculty of Medicine Prince of Wales Hospital The Chinese University of Hong Kong Shatin Hong Kong China; ^5^ Department of Orthopaedics and Traumatology Li Ka Shing Institute of Health Sciences Faculty of Medicine Prince of Wales Hospital The Chinese University of Hong Kong Shatin Hong Kong China

**Keywords:** artificial ligament, biomimetic film, osseointegration, tissue regeneration, translational potential

## Abstract

Control over soft‐to‐hard tissue interfaces is attracting intensive worldwide research efforts. Herein, a bioactive film‐guided soft–hard interface design (SHID) for multi‐tissue integrative regeneration is shown. Briefly, a soft bioactive film with good elasticity matchable to native ligament tissue, is incorporated with bone‐mimic components (calcium phosphate cement, CPC) to partially endow the soft‐film with hard‐tissue mimicking feature. The hybrid film is elegantly compounded with a clinical artificial ligament to act as a buffer zone to bridge the soft (ligament) and hard tissues (bone). Moreover, the bioactive film‐decorated ligament can be rolled into a 3D bio‐instructive implant with spatial‐controllable distribution of CPC bioactive motifs. CPC then promotes the recruitment and differentiation of endogenous cells in to the implant inside part, which enables a vascularized bone growth into the implant, and forms a structure mimicking the biological ligament–bone interface, thereby significantly improving osteointegration and biomechanical property. Thus, this special design provides an effective SHID‐guided implant‐bioactivation strategy unreached by the traditional manufacturing methods, enlightening a promising technology to develop an ideal SHID for translational use in the future.

## Graphical abstract

A elastomeric film is bioactivated by calcium phosphate cement (CPC)‐incorporation, which is then compounded with clinical artificail ligament to form a soft‐hard‐interface mesh. The mesh can be rolled into a 3D bio‐instructive implant with spatial‐controllable distribution of CPC, a structure efficiently facilitates multi‐tissue integrative regeneration by inducing vascular and new bone ingrowth.

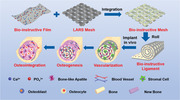

## 1 Introduction

The rational design of soft‐to‐hard material interfaces has been attracting accumulating attentions in the fields of tissue engineering and material science.^[^ ^1^ ^]^ The integration of multiscale tissues, especially the functional soft‐to‐hard interface integration plays a vital role in both artificial device development (e.g., skin‐conformal electronic devices) and biomedical engineering (e.g., interface tissue engineering to facilitate the reconstruction/regeneration of connective tissues).^[^ ^1^, ^2^ ^]^ A flexible and stable integration between soft and hard material interfaces is difficult to achieve due to the different mechanical properties, which remains a challenge in material design/development.^[^ ^1^ ^]^ Especially, the difficulties on soft‐to‐hard interfacial tissue reconstruction (e.g., cartilage and bone) result in major clinical problems,^[^ ^2^ ^]^ which significantly impact the life qualities of large numbers of people suffering from tissue or organ failure after musculoskeletal injuries, such as damage to the connection between anterior cruciate ligaments (ACL) and bones.^[^ ^3^ ^,^ ^4^ ^]^

Soft‐to‐hard tissue interfaces (e.g., ligament‐to‐bone, tendon‐to‐bone, etc.) exhibit anisotropic structural properties, which gradually change from one tissue to another, and especially, the translational region between soft connective tissue and bone plays a key role in the mechanical load transmission from soft to hard tissues by reducing the interfacial stress.^[^ ^5^, ^6^, ^7^ ^]^ The regeneration failure at this region often results in a poor mechanical transfer at the interface to cause a serious re‐injury.^[^ ^8^, ^9^, ^10^ ^]^ Current surgical approach mainly uses autograft or allograft, which has been reported to have a high re‐injury rate of up to 30% within the first year after surgery,^[^ ^11^ ^]^ mainly associated with the poor bridging structure undergoing ligamentization with necrosis, limited revascularization and low‐quality remodeling, during which the graft strength decreases dramatically.^[^ ^12^, ^13^, ^14^ ^]^ In addition, the autograft and allograft have other disadvantages, like limited resources, unavoidable tissue injury, inflammatory response, as well as immune rejection,^[^ ^11^, ^15^, ^16^ ^]^ which calling the need for advanced artificial replacements. Although synthetic materials (e.g., the clinically used ligament advanced reinforcement system, LARS) show the advantage of a high and non‐reduced strength than autograft,^[^ ^17^ ^]^ its poor integration with bone results in unqualified mechanical fixation with host bone, which is due to the lack of bioactive components^[^ ^18^, ^19^ ^]^ and results in the lack of cell affinity of biomaterial (which impairs the recruitment and adhesion of tissue‐repairing progenitor cells), the insufficient neovascularization of the implants, and the retarded osteogenic differentiation of progenitor cells (e.g., bone marrow stromal cells, BMSCs), hindering bone‐in‐growth and thereby reducing the integration with bone (termed as osseointegration).^[^ ^19^, ^20^, ^21^, ^22^, ^23^, ^24^ ^]^ Therefore, it is critical to find an approach to vitalize the synthetic ligament to enhance the osteointegration and achieve intrinsic bio‐fixation, a strategy to facilitate soft‐to‐hard interface tissue regeneration.

The bone‐mimicking features of the calcium‐phosphate (CaP) materials (accounting for 70% in natural bone tissue) make them as the ideal bioactive candidates for regulating bone cell behaviors (guided recruitment and osteogenic differentiation) and bone regeneration functions.^[^ ^25^, ^26^, ^27^, ^28^, ^29^ ^]^ Especially, calcium phosphate cement (CPC), a highly promising bone substitute developed by our group,^[^ ^31^, ^32^, ^33^ ^]^ has been clinically used in more than one million clinical cases, and thereby can serve as bone‐mimic components for soft material modification. By introducing CPC components onto the inert ligament material (which can effectively connect with host soft tissue), an ideal soft‐to‐hard interface could be potentially achieved through improvement of a CPC‐induced osteointegration. However, it is difficult to introduce sufficient amount of CaP bioactive components on the organic polymer‐based implants due to the brittleness of inorganic materials themselves. Additionally, the weak binding of CaP particles to implant often results in a high chance of peeling off, leading to complications such as inflammatory arthritis.^[^ ^19^, ^30^ ^]^ Ideally, sufficient bioactive elements should be rationally compounded with implant to ensure a bone in‐growth throughout the implant network to effectively enhance osteointegration.

**Scheme 1:**
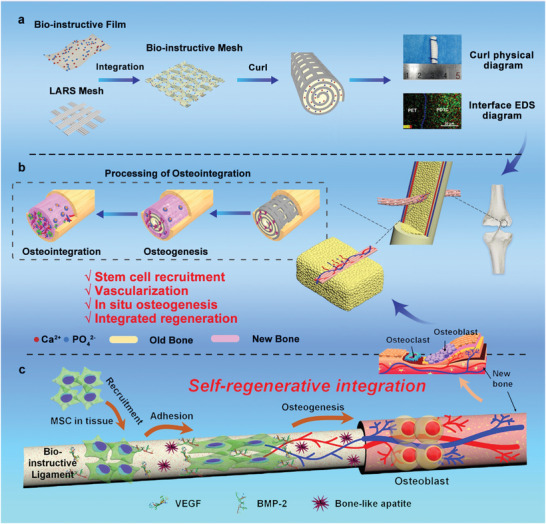
Schematic representation of the mechanisms underlying the film‐guided soft–hard interfacial design for multi‐tissue integrative regeneration. a) The film‐aiding processing technology allows for an introduction of sufficient amount of calcium phosphate cement (CPC) with controllable spatial distribution onto an elastic bioactive film of poly(lactic acid‐carbonate) (PDT), and meanwhile preserves a microscopic structure which facilitates the in situ generation of bone‐like apatite structure to endow the LARS ligament with bionic features (bone formation bioactivity) in vivo. b) The bioactive film could be easily rolled to form the ligament for clinical use, with its macroscopic porous structure to facilitate osteointegration by attracting blood vessel and new bone ingrowth to the inside part of ligament along with CPC distributed on the film. c) Cellular and molecular mechanisms of the bio‐instructive ligament induced osteointegration. CPC on the film can spontaneously recruit endogenous stem/stromal cells and induce their angiogenic and osteogenic differentiation by upregulating the production of host‐derived bioactive motifs (VEGF, BMP 2, etc.), and thereby subsequently facilitating the ligament–host bone integration via accelerating neovascularization and new bone formation at both the periphery and the center of the ligament.

Herein, we develop a film‐guided soft–hard interfacial design (SHID) to synthesize a bio‐instructive ligament with spatial‐controllable distribution of bioactive components. To facilitate soft‐to‐hard tissue integration, first, we chose a bioabsorbable polymer (lactide‐co‐trimethylene carbonate, PDT, a bioabsorbable polymer material for fabrication of artificial blood vessel in clinical application^[^ ^34^, ^35^ ^]^) as a soft material matrix to ensure the connection with host ligament, due to its good mechanical elasticity. Then, bone‐mimicking CPC, is hybridized with PDT (to form PDT‐CPC, PDTC) to endow PDT with hard tissue mechanical features. The PDTC membrane was then combined with LARS to form a combined bioactive mesh with proper distribution of bioactive components, providing a microscopical structure to benefit osteointegration (due to CPC‐induced osteogenesis). After that, the bioactive mesh could be handily rolled to transform from a 2D membrane to a 3D ligament implant, where its native macroscopic porous structure with spatially‐distributed CPC on the film could benefit self‐biomineralization to attract stromal cells from the outside to migrate into the inside of ligament, subsequently promoting their angiogenic and osteogenic differentiation. Meanwhile, the porous structure owned in the LARS mesh facilitates cell migration and growth inside the ligament (improving nutrition/waste exchange).^[^ ^36^ ^]^ Thereby, our design could facilitate the host bone growth into the implant inside part, forming a structure mimicking the biological ligament–bone interface, hence significantly improving osteointegration and biomechanical property. Furthermore, with the gradual biodegradation, Ca and P elements could be sustainably released, which would regulate the local microenvironment to facilitate angiogenesis, osteogenesis, and ECM construction, a process further contributing to the bionic regeneration of ligament and its integration with bone. The specially designed implant effectively guides an outside‐to‐in bone growth and enhances bone‐graft osteointegration via promoting multi‐tissue reconstruction through optimization of its special structure and biomimetic functions (**Scheme** **1**). The current study therefore provides a bioactive film‐guided SHID for multi‐tissue integrative regeneration.

## 2 Results and Discussion

**Figure 1:**
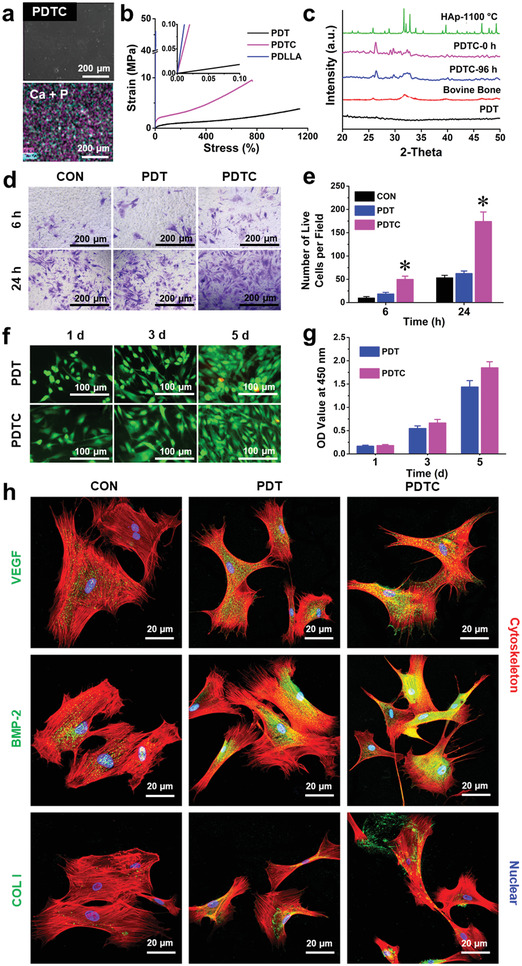
Physiochemical properties and in vitro biological assays of the bio‐instructive films. a) Energy dispersive spectrometry (EDS) analysis of the hybrid film. b) Stress–strain curves of PDLLA, PDT, and PDTC films. c) X‐ray diffraction (XRD) analysis of the hybrid film before and after biomineralization (96 h incubation in SBF buffer), the native bovine bone, and the sintered HAp particles.^[^ ^36^ ^]^ d,e) Transwell migration assays of BMSCs cultured on the PDT and PDTC films, cells cultured on blank cell dish served as control. f,g) Cell proliferation assays of BMSCs cultured on PDT and PDTC films. h) Protein expression of VEGF, BMP‐2, and Col I in the BMSCs after 7 days incubation with the PDT and PDTC films (blank cell dish served as control). (^*^ *p* < 0.05 compared to the control group, *n* = 3).

Due to the poor interfacial weak binding state, the traditional physically‐coated bioactive components tend to peel off from the LARS ligament to cause severe inflammatory arthritis.^[^ ^37^ ^]^ To solve this problem, we first developed a bioactive component‐incorporated biomimetic biodegradable film, which could then strongly bind with LARS and thereby introduce sufficient amount of bioactive components (CPC, to deliver Ca and P compositions favorable for osteogenesis and BMSCs recruitment^[^ ^25^, ^26^ ^]^) on the artificial ligament, an advanced strategy to enhance the osteointegration capacity. To overcome the brittleness of the traditional poly (D, L‐lactide) (PDLLA) material, poly (lactide‐co‐trimethylene carbonate) (PDT, FDA‐approved materials) with good elasticity was synthesized via a ring‐opening polymerization technique in our lab. The resulting polymer presented NMR peaks at 1.60 and 5.15  ppm corresponding to ─CH_3_ unit and ─CH unit on the LA chain segment, peaks at 2.10 and 4.30 ppm^[^ ^34^ ^]^ corresponding to ─CH_2_‐O unit and ─CH_2_─ unit on the trimethylene carbonate (TC) chain segment (Figure S1, Supporting Information), which could be used for calculation of LA/TC mole ratio as 70:30. The molecular weight of PDT is 364.2 kDa based on the GPC analysis (Table S1, Supporting Information), and its high molecular weight allows for the formation of a bioabsorbable film with mechanical robustness. After that, CPC powders were hybridized with the PDT polymer through a mild blending technique which enabled the homogenous distribution of the bioactive CPC components (e.g., Ca and P) into the films (**Figure** **1**a). After optimization, we obtained a series of CPC‐doped films. The optimal film could be stretched to (740  ± 41)% of its original length (Figure 1b), while the deformed films could be recovered to its original shape in 30.0 s under biological temperature atmosphere (Figure S2, Supporting Information), suggesting its robust flexibility and excellent elastic memory functions. The Young's modulus of PDT is closer to that of native ligament tissue^[^ ^38^ ^]^ (Figure S3, Supporting Information). After adding CPC, the Young's modulus of PDTC active membrane increases significantly compared with that of PDT (Figure S3, Supporting Information), and its brittleness has been greatly improved compared to PDLLA (Figure 1b). After the introduction of CPC, the bioactivity of the inert PDT membrane was improved greatly (Figures S4 and S5, Supporting Information), as demonstrated by the induced bio‐mineralization and cell adhesion in PDTC membrane. Moreover, as CPC can be transformed into the bone matrix through biomineralization in vivo into bone‐like apatite (a pre‐requisite for differentiation of hMSCs^[^ ^39^ ^]^), the mineralized composite membrane showed a bone‐mimicking crystalline structure similar to natural bone tissue, which was different from the conventionally‐sintered HAp particles with high crystallinity difficult to be bio‐absorbed for bone regeneration (Figure 1c). The hybrid film can deliver Ca and P ions to improve cell recruitment, and CPC‐directed biomineralization should be beneficial to guide a directional osteogenic differentiation for bone regeneration.^[^ ^40^ ^]^ The mechanical robustness and shape memory intelligence, together with its excellent biodegradability (Figure 1b; Figures S2 and S11, Supporting Information), enable SHID the potential as an ideal tool for inert implant modification to provide bioactive functions, thereby to develop advanced biomaterials such as PDTC hybrid film.

The interactions between host cells and biomaterials are considered to play decisive roles in tissue engineering. It is indicated that the recruitment of endogenous host cells into implants is a determinant procedure for tissue regeneration in vivo.^[^ ^21^, ^41^ ^]^ As the bioactive inorganic natural component, calcium‐phosphate can promote the absorption of endogenous growth factors (e.g., bone morphogenetic protein (BMP)) favoring to osteogenesis in vivo.^[^ ^42^, ^43^ ^]^ It was thus proposed that doping CPC into the membrane might facilitate the host cell recruitment for bone regeneration. To verify this, the effects of the PDT and PDTC films on BMSCs recruitment were evaluated using transwell migration assay (the blank cell dish substrate was set as a negative control). Similar to the control group, few BMSCs migrated toward PDT film after 6 h of incubation. In comparison, BMSCs numbers significantly increased (*p* <  0.05) in response to PDTC film (Figure 1d,e). After 24 h culture, the cell numbers recruited by PDTC film was 3.5‐folds to that of PDT film, demonstrating that CPC hybridization significantly enhanced host progenitor cell recruitment. To examine the effect of CPC‐associated immune microenvironment on BMSCs recruitment,^[^ ^44^ ^]^ we seeded macrophages (RAW264.7 cells) on the material surface, and then used transwell to co‐culture with BMSCs. The migration of BMSCs was evaluated, and the results showed that PDTC group still induced a good cell recruitment (Figure S6, Supporting Information), suggesting that PDTC could coordinate with immune cells (macrophages) to generate a microenvironment favoring BMSC recruitment, a key factor to initiate a quick bone regeneration.^[^ ^45^ ^]^

To evaluate the effects of the hybrid films on cell survival and growth, live/dead staining and CCK‐8 assay were performed to examine BMSCs cultured on pure polymer film (PDT) and hybrid film (PDTC). As shown in Figure 1f, from day 1 to day 5, BMSCs (green) continuously proliferated on both PDT and PDTC films, and the cell viability on PDTC film was comparable to that on the PDT film (Figure S7, Supporting Information). Both PDT and PDTC can promote cell proliferation (Figure 1g). The effects of the films on the expression of functional factors regarding angiogenesis/osteogenesis associated (e.g., VEGF (an important angiogenic index^[^ ^46^ ^]^) and BMP‐2 (an important osteogenic factor^[^ ^47^ ^]^) expression, Col I (extracellular matrix of bone^[^ ^48^ ^]^) secretion) were detected via immunofluorescence (IF) staining and ELISA. As shown in Figure 1h and Figure S8, Supporting Information, cells cultured on the PDTC film showed the highest expression and secretion of VEGF, BMP‐2, and Col I. This is probably due to the CPC, which could effectively promote osteogenesis, and increased the expression of VEGF correspondingly.^[^ ^49^ ^]^ Besides, the hybridization of CPC into PDT may increase the film's hydrophilicity and thus cooperatively promote its cell affinity, proliferation, and osteogenic differentiation.^[^ ^50^, ^51^ ^]^ The higher osteogenesis induced by the hybrid film could be further supported by its enhanced alkaline phosphatase (ALP) and Alizarin red staining intensity, as compared to the native polymer film (PDT) (Figure S9, Supporting Information).

**Figure 2:**
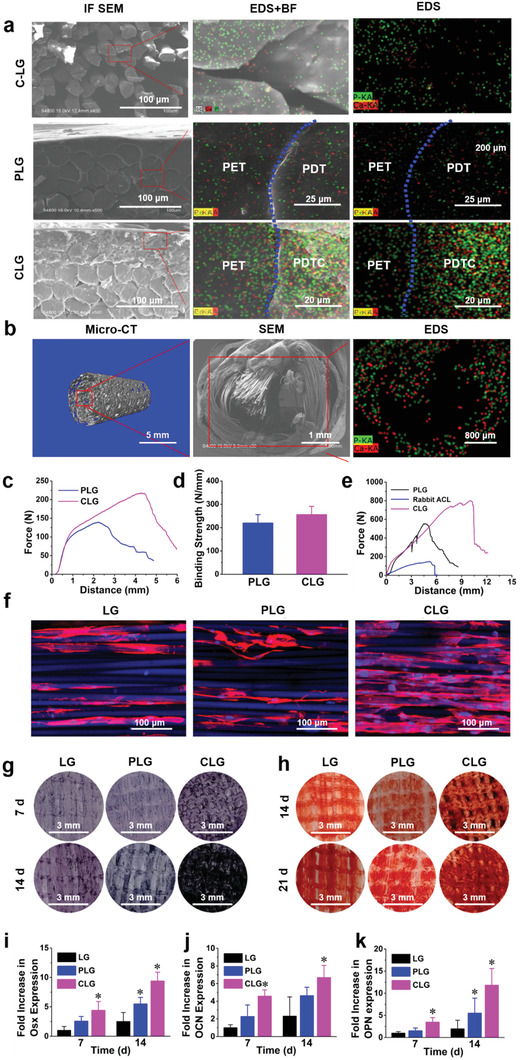
In vitro biological assays of the ligament graft. a) Scanning electron microscope (SEM) and energy dispersive spectrometry (EDS) analysis of the interface between ligament and membrane, IF: interface; BF: bright field. b) The micro‐CT, SEM, and EDS analysis of the spatial distribution of CPC components. c,d) The binding force and binding strength of PDT/PDTC coating on PLG/CLG. e) The tensile force–distance curve of rabbit ACL, PLG, and CLG. f) Cell adhesion of BMSCs cultured on the PLG and CLG ligament grafts for 4 days, with LARS ligament (LG) as control. g) Alkaline phosphatase (ALP) staining of differentiating BMSCs cultured on PLG and CLG ligament grafts after 7 and 14 days, with LG as control. h) Mineralization of BMSCs cultured on PLG and CLG ligament grafts after 14 and 21 days with LG as control. i–k) Expression of Osx, OCN, and OPN in BMSCs after 7‐ and 14‐days culture on PLG and CLG ligament grafts with LG as control. (^*^ *p* < 0.05 compared to the control group, *n* = 3).

To retain the biological functions of the hybrid film, we developed a thermo‐compression technique to assemble CPC‐doped film into artificial ligament mesh under 80  ℃, to generate the CPC‐doped ligament graft (CLG). By tuning the film building‐block composition and manufacturing process, we were able to generate a diverse set of assembled ligament–bone integrated graft with bioactive properties. PDT modified ligament (PLG) and pure LARS treated with CPC solution (C‐LG) served as controls. The flexibility of the film preserves the integrity of the graft native porous structure through a simple rolling procedure, which ensures a controlled spatial distribution of CPC from the outside to inside of CLG. (**Figure** **2**a; Movie S1, Supporting Information). The results showed that CPC was combined with the LARS mesh like a flat cloth, which distributed evenly on the mesh. After crimping the mesh, CPC distribution from outside to inside of the graft could be observed (Figure 2b; Movie S2, Supporting Information). Both, the EDS results showed that the introduction of Ca and P in CLG was much better than C‐LG, as well as the amount of input and the spatial distribution of CPC (Figure 2a). Therefore, we chose CLG as the implant material instead of C‐LG in the following study. Moreover, the introduction of PDTC film did not change the grid structure of the original mesh, and the original porous structural characteristics were retained (Figure S10, Supporting Information). The film enabled a controllable biodegradation process, and allowed for a sustainable delivery of bioactive ions (calcium, phosphate) into the local microenvironment, which is beneficial to bioactivate the LG to facilitate the cell recruitment, migration, adhesion, and differentiation for tissue regeneration (Figure S11, Supporting Information). It is worth mentioning that the mass loss of the CPC containing group (PDTC and CLG) is smaller, which is supposed to be due to the water absorption of CPC mineralization. The remained PDTC and CLG grafts were scanned by EDS again. The results showed that there are still a big amount of Ca and P components in both PDTC and CLG (Figure S12, Supporting Information).

The binding forces of PDT film and PDTC film with LARS mesh were (127.0  ± 6.9) N and (218.0  ± 9.5) N (*p* < 0.01) (Figure 2c), respectively. The binding strengths of them were 219.7  ± 20.8 and 255.7  ± 20.5 N mm^−1^ (Figure 2d). The binding forces were in accordance with the cross section SEM of grafts (Figure 2a). From our results, it could be observed that there was a stronger binding between PDTC and LARS mesh (as compared with PDT), indicating that our technology could effectively introduce a firm binding of CPC component to LARS to endow it with osteogenic bioactivity. The mechanical property of artificial ligament is indispensable in ligament reconstruction, which should be sufficient for daily movement/sports. The inferior mechanical property of auto‐ or allo‐graft is the major challenge in clinical use. In this study, the failure force of CLG was 1.3 times of PLG and 5.0 times of rabbit autogenous ligament (Figure 2e), suggesting the excellent mechanical property of CLG, which was much better than the autograft (rabbit ligament), and thereby should be a suitable artificial LG.

The adhesion of BMSCs on LGs was consistent with that of composite membrane (Figure 2f; Figure S5, Supporting Information). Due to the smooth and hydrophobic surface, the cell affinity and cell density on PLG were similar to that on the LARS LG. Comparatively, CLG showed stronger cell affinity, allowing for more cell attachment and spreading along the ligament fibers with good orientated cytoskeleton morphology (Figure 2f). The ALP staining results of differentiating BMSCs (on 7 and 14 days) also showed that CLG induced the highest ALP expression intensity (Figure 2g), indicating that CLG should promote the best early osteogenic differentiation of BMSCs. Accordingly, CLG group also displayed an enhanced Alizarin red staining intensity than the PLG and LG groups at both day 14 and 21, indicating a promoted late‐stage mineralization associated with the CPC hybridization (Figure 2h). qPCR was then performed for quantitative osteogenesis‐related gene analysis. The results indicated that the CLG induced the highest mRNA levels of osteogenic markers osterix (Osx, involved in the regulation of osteoblasts^[^ ^52^ ^]^), osteocalcin (OCN, responsive for the regulation of mineral secretion^[^ ^53^ ^]^), and osteopontin (OPN, a structural protein synthesized by osteoblasts and osteocytes^[^ ^54^ ^]^) (Figure 2i–k) in differentiating BMSCs. Overall, the CLG presented the best osteogenic differentiation ability. As such, bioactive multi‐tissue artificial ligament–bone implant with good flexibility was successfully constructed. The ligament bioactivation technology may allow for the introduction of sufficient bioactive components with functional bionics into the ligament, thus enhancing the interfacial bone formation to effectively improve the ligament–bone osteointegration.

**Figure 3:**
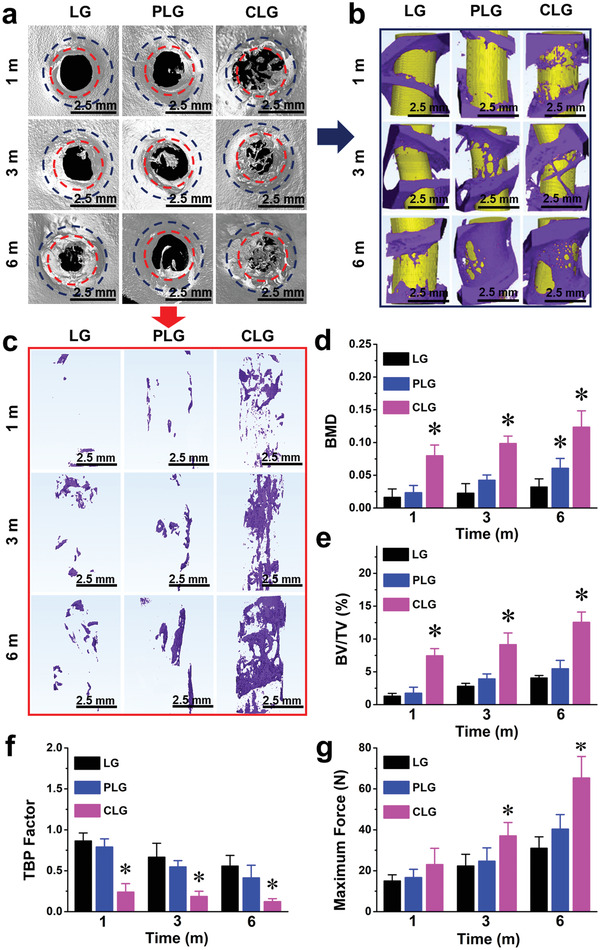
Evaluation of bone regeneration and osseointegration with the ligament grafts in vivo. a) 3D reconstruction of micro‐CT scanning of host bone with grafts. b,c) Marginal and internal new bone formation in rabbit tibia implanted with the PLG and CLG ligament grafts after 1, 3, and 6 months, with LARS ligament (LG) as control. d–f) Bone mineral density (BMD), bone volume density (BV/TV), and trabecular pattern factor (TBPf) of rabbit tibia implanted with PLG, CLG, and LG ligament grafts after 1, 3, and 6 months. g) The maximum osseointegration tensile forces after implantation of PLG, CLG, and LG ligaments into rabbit tibia for 1, 3, and 6 months. (^*^ *p* < 0.05 compared to the control group, *n* = 3).

As CLG showed potential osteoinductive capacity in vitro, further in vivo experiments were performed to validate whether it could induce bone formation and improve graft osteointegration. Regarding its structure and mechanical properties, CLG mimicked the normal tendon‐bone insertion with high tensile strength (Figure 2e) and thereby was considered to induce in situ bone regeneration, to facilitate graft–bone osseointegration and thus lead to biofixation of artificial ligament into bone tissue. To verify the hypothesis, cylindrical grafts (LG, PLG, and CLG) with a size of 2.0  cm length and 2.5  mm diameter were implanted into a rabbit proximal tibial tunnel, a widely used graft–bone healing model for ACL reconstruction study.^[^ ^55^, ^56^ ^]^ The results of new bone formation and graft–bone integration were analyzed via radiography, biomechanics, and histochemistry assays. The new bone morphologies at month 1, 3, and 6 post surgery, were examined by micro‐CT and analyzed using 3D reconstruction technique (**Figure** **3**a). Limited new bone formation (mainly restricted at graft margins) could be observed in LG group at month 3 and 6 (Figure 3a,b), suggested no obvious osteoinductivity with LARS LG. The results of PLG group were similar to that of LG group at 1 month, and new bone formation increased significantly around the graft at month 3 and 6 (Figure 3b), while seldom new bone formation could be observed inside the graft (Figure 3c). The results indicated that the osteoinductivity of PDT should be insufficient to promote bone regeneration inside the graft. On the other hand, in CLG group, new bone formation could be observed at the graft margins at month 1 and increased significantly from month 3 to 6 (Figure 3a). Furthermore, within the CLG, lots of scattered new bone were observed at month 1, and sufficient new bone with block‐like structure was formed and grew significantly from month 3 to 6 (Figure 3c; Movie S3, Supporting Information). The micro‐CT scanning of single layer from different plates (sagittal, coronal, and transverse plates) (Figure S13, Supporting Information) further demonstrated the new bone formation throughout the whole defect area. The statistical analysis results (Figure 3d–f) were in accordance with the above results. Compared to the LG and PLG groups, bone mineral density and bone volume/total volume (BV/TV) of CLG group markedly increased, while trabecular pattern factor (TBPf) markedly reduced. Notably, the biomimicking features of the PDTC film could markedly improve the bioactivity of multi‐tissue grafts without the need for any extra cytokines or growth factors.

The final purpose of a satisfactory graft–bone integration is to enhance the graft biomechanical property in the bone tunnel, via improving the load‐to‐failure property, which is vital for ligament reconstruction to transfer the dynamic stress between soft and hard tissues. The maximum tensile forces of three grafts in vivo were examined (Figure 3g; Figure S14, Supporting Information). At month 1, the failure force of CLG group was 23.0  ± 4.6 N, which was higher than those of PLG (16.7  ± 2.3 N) and LG groups (15.0  ± 1.7 N). At month 3, the maximum force of CLG group increased by 60% to 37.0  ± 3.8 N, which was significantly higher than those of PLG and LG groups. At month 6, the maximal force of the CLG increased sharply to reach 65.3  ± 6.1 N, which was almost two folds of the LG group. According to the previous research,^[^ ^57^ ^]^ in 3 months after ACL reconstruction, the failure force of autologous ligament and decellularized allogenic tendon were 18.6  ± 7.3 and 27.6  ± 8.8 N, respectively. These values are significantly lower than the failure force of CLG at the same period in the current study (Figure 3g), indicating that CLG can accelerate osteointegration than the natural ligament.

**Figure 4:**
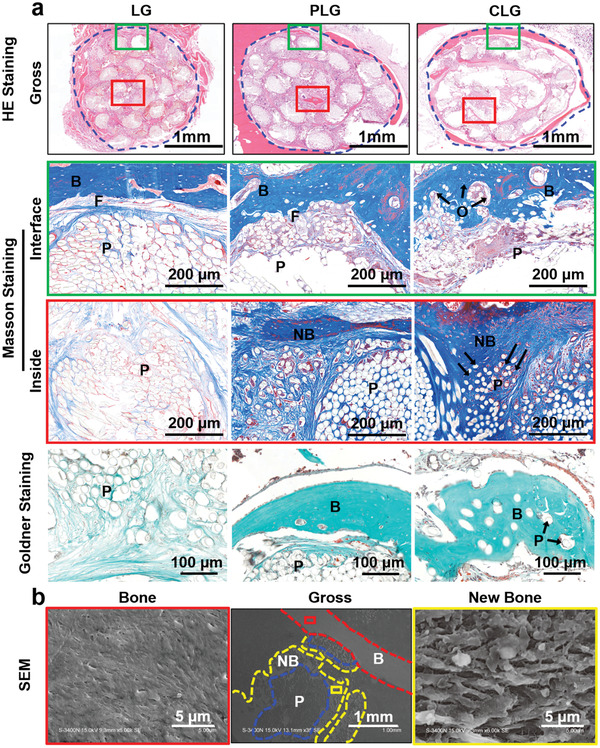
Histological analysis of tissues with implanted ligament grafts in vivo. a) The HE, Masson, and Goldner staining of the ligament (PLG, CLG, and LG)‐bone after 6 months post implantation, the dotted blue circle indicates the implanted grafts. b) The SEM photos of the CLG ligament–bone after 6 months after implantation. (P: PET; F: Fibrous interface; B: Bone; NB: New Bone; O: Osteoid; the black arrow indicates graft–bone osteointegration interface).

**Figure 5:**
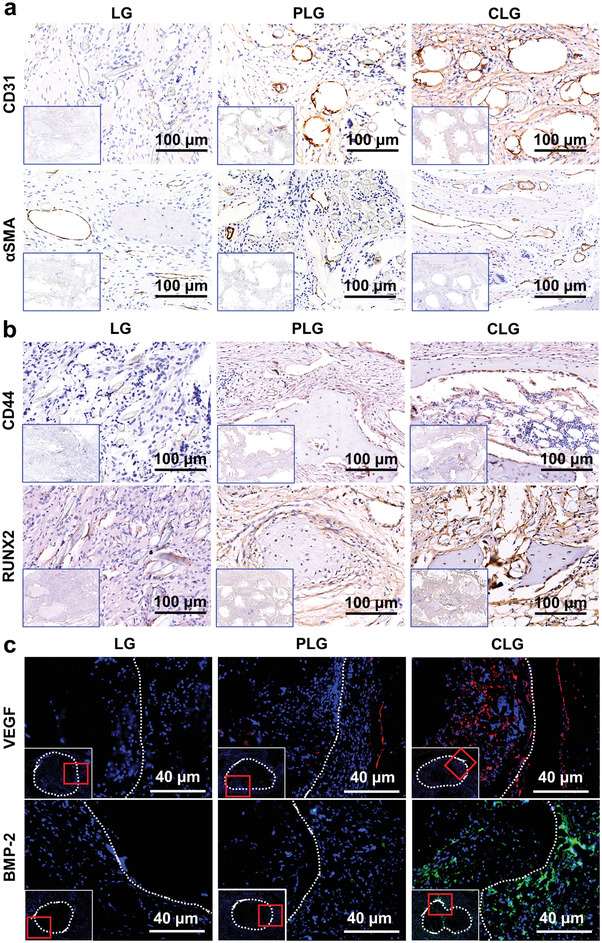
Expression of the angiogenesis and osteogenesis markers in the implanted ligament associated tissue. a,b) The immunohistochemistry (IHC) staining against CD31 (1 month), *α*SMA (1 month), CD44 (1 month), and RUNX2 (3 months) and of ligament (PLG and CLG)‐bone interface, with LARS ligament (LG) as control. c) The expression of VEGF at 1 month and BMP‐2 at 3 months in ligament (PLG and CLG)‐bone interface, with LARS ligament (LG) as control.

Histological analysis was then performed to detect whether CLG could enhance the graft–bone integration and thus improve the biomechanics (procedure described in Figure S15, Supporting Information). As shown in **Figure** **4**a and Figure S16a, Supporting Information, in LG group, even after 6 months, no new bone formation inside the grafts could be observed, and only a small amount of new bone was formed in the surrounding area. In comparison, PLG group induced obvious new bone formation around the graft and few new bone inside the graft until month 6. In CLG group, new bone formation was observed both around and inside the graft in the early stage and increased thereafter (Figure 4a; Figure S16a, Supporting Information). SEM results showed that the structure of bone inside CLG was significantly different from that of the external host bone (mature bone), indicating that the internal bone should be new bone (Figure 4b; Figure S17, Supporting Information). Masson staining,^[^ ^58^, ^59^ ^]^ a specific staining for collagen fibers, was performed to investigate the graft–bone interface integration and intra‐graft bone regeneration, which can distinguish the mature bone (red) from the immature bone (less red).^[^ ^60^ ^]^ Both LG and PLG groups displayed a fibrous non‐integration interface at each time point, while the width of the graft–bone interface in PLG group was significantly reduced as compared with LG group (Figure 4a; Figures S16b and S18a, Supporting Information). In CLG group, PET fibers were directly integrated with osteoid tissue assembling the clustered and active cells at the interface at month 1 and 3. Direct integration of graft and new bone was observed at month 6 (Figure 4a; Figures S16b and S18b, Supporting Information), and intra‐graft bone regeneration then occurred (Figure 4a; Figure S16c, Supporting Information). No new bone could be observed in LG group at each time point. At month 1, there were lots of new vessel‐like structures, which could facilitate cell migration and nutrient transport in PLG group. Meanwhile, dense fibers and osteoid formation around the blood vessel‐like structures were observed in CLG group. At month 3, the scattered new bone could be observed in PLG group, while new bone and PET fibers in CLG group formed osteointegration at this time point. At month 6, the new bone was directly integrated with PET fibers to form firm osteointegration in CLG group (Figure 4a; Figures S16c and 18c,d, Supporting Information). To further verify the possible integration between new bone and grafts, Goldner staining was performed, and as shown in Figure S16d, Supporting Information, in 1‐month post‐implantation, there were lots of BMSC‐like cells around CLG, suggesting that CLG should has the ability to recruit endogenous stem cells. Further results showed that the CLG graft significantly facilitated the recruitment and migration of CD44^+^ bone marrow mesenchymal stem cells toward the scaffolds, and the induced RUNX2 expression demonstrated that CLG could promote the recruited stem cells to differentiate into RUNX2+ osteoblasts (**Figure** **5**; Figure S19, Supporting Information). It is well recognized that endogenous stem cells play an important role in bone repair,^[^ ^61^ ^]^ and therefore, this result further demonstrated that CLG should have excellent bone regeneration capacity. While for PLG, only large numbers of red blood cells could be observed. In 3‐month post‐implantation, the new bone formation occurred in both CLG and PLG groups, while CLG group showed more new bone than PLG group. After 6‐month post‐implantation, no new bone could be observed in the two groups, suggesting that new bone formation should be accomplished at this stage. Integration between bone tissues (green areas) with ligament fibers could be tracked inside CLG, while only surrounding bone tissue could be seen in PLG. These results indicate that CLG could effectively induce new bone formation in situ, which is integrated with the PET fibers firmly along with the degradation of bioactive composition, thus improving osteointegration and biomechanical function.

Currently, connective tissue reconstruction remains as a major challenge, particularly the regeneration at the soft‐to‐hard tissue interface.^[^ ^7^, ^9^, ^10^, ^11^, ^12^ ^]^ To enhance soft‐to‐hard tissue integration, a strategy is to add in hard‐tissue mimic into the soft material; moreover, the soft material could be modified to facilitate the integration with hard tissue. Thereby, in the current study, we developed a bioactive film‐guided SHID, by simply adding in bone‐ mimic component CPC onto PDT, a soft material with a mechanical property similar to the biological ligament, to form a bioactive membrane (PDTC) to combine with the clinical‐used ligament material (LARS), which not only endows the hard‐tissue feature into LARS, but also significantly improves the integration with host bone by facilitating host bone ingrowth into the ligament material.

The poor host tissue integration with the artificial material (LARS) is mainly due to the reason that the inert material lacks bioactive components, therefore is unable to form a biochemical combination with the host tissue. A previous strategy is to introduce bioactive component onto the surface of the artificial ligament by physical coating and impregnation.^[^ ^62^, ^63^ ^]^ However, such approaches are unsatisfactory due to the low loading efficiency and poor binding with the components, which usually results in insufficient ligament integration and inflammatory response.^[^ ^19^, ^34^ ^]^ To solve these problems, in this study, we developed a technology (SHID) with the novel design utilizing a bioactive elastic membrane and mild composite method, to achieve the stable loading of large‐scale bioactive substances, on the surface of the inert ligament (LARS). This technology therefore first‐timely provides an advanced approach to bioactivate the artificial ligament with components other than CPC (e.g., nanoparticles to deliver cytokines/drugs) in the future, which will significantly improve the development of artificial ligament for clinical use. The SHID could also potentially be used to modify material types other than ligament to facilitate the soft‐to‐hard tissue integration in the future, which still needs further investigation.

From the material point of view, the current study also provides an advanced ligament material for potential translational use. By simply rolling, our material could be elegantly transformed from a 2D membrane into a 3D ligament, with controllable spatial distribution of CPC from the surface into the inner parts. CPC could efficiently facilitate the recruitment of progenitor cells and guide them to migrate into the artificial ligaments, accelerate angiogenesis and osteogenic differentiation (as indicated with our in vivo results), hence significantly facilitate new bone growth into the ligament following CPC distribution, an effect benefiting self‐regenerative integration. Accordingly, our in vivo experimental results have shown that this active artificial ligament could significantly induce osteointegration and improve biomechanics through a biochemical fixation between material and bone (Table S2, Supporting Information). Furthermore, the in situ mineralization of CPC could form an osteoid bionic function, which, together with the preserved porous structure of LARS mesh, should be beneficial for cell adhesion, nutrient transportation, and the ingrowth of small blood vessels with reduced foreign body response (Figure 5; Figure S21, Supporting Information). This thereby facilitated the formation of a forming a material‐host bone interfacial structure mimicking the natural interface between host ligament and bone, hence significantly improving osteointegration and biomechanical property. Besides, the mechanism of osteoinduction by CLG could be attributed to the reasons that PDTC could constantly deliver calcium and phosphorus during biodegradation process, and adsorb endogenous growth factors (including VEGF, BMP‐2) from the body fluids, which together facilitate the recruitment and differentiation of endogenous progenitor cells to form new bone.

This study partially reveals the materiobiology principles in biomaterials design for tissue regeneration.^[^ ^45^ ^]^ First, at the cell‐level, CPC with bone‐mimicking hydroxyapatite transformation potential successfully promoted the migration of the recruited progenitor cells onto biomaterials through the release of specific biophysical/chemical signals, and then stimulated the cells to differentiate in the direction of osteogenesis.^[^ ^64^ ^]^ Biomineralized structure of CPC and appropriate mechanical properties of elastic membrane can enhance cell adhesion and proliferation.^[^ ^65^, ^66^ ^]^ Second, at the tissue level, biomaterials can process the bionic function. The in situ mineralized apatite mimics bone‐like structure, which, in combination with the PDT, facilitated the absorption of endogenous cytokines and growth factors to induce vascularization and facilitate tissue regeneration.^[^ ^34^, ^67^ ^]^ Moreover, at the multi‐tissue level, biomaterials lead to a graft–bone integrated repair. The artificial implant was designed with a structure to attract endogenous cells to migrate inside, where they could start differentiation to facilitate graft–bone osteointegration. Therefore, the appropriate active components and well‐designed structures effectively introduced the bone growth into the graft inside, enhanced the integrating force between ligaments and bone, which drastically improved the integrated repair of soft and hard tissues.

## 3 Concluison

To sum up, the current study successfully develops bioactive SHID for multi‐tissue integrative regeneration, by using a versatile film‐guided technology to bioactivate the inert ligament implant, with the novel design that bioactive ingredients can be assembled into a robust biodegradable film, and then the film of controlled size and specific bioactive element amount can act as a functional unit to be compounded with the inert ligament mesh under mild compression approach. The resulting film‐modified ligament mesh can be rolled to form a bio‐instructive artificial ligament implant with well‐defined spatial distribution of bioactive ingredients for multi‐tissue regeneration. This bioactive implant can create an ideal microenvironment for endogenous progenitor cells recruitment and differentiation, thereby stimulating a powerful and scalable self‐regenerative integration. Moreover, the material could release osteoinductive elements during the biodegradation process, which, together with its improved biomineralization capacity, effectively facilitated the vascularized bone regeneration. Hence, this bio‐instructive ligament would greatly improve osteointegration with bone via attracting bone ingrowth, which has been demonstrated by our in vivo results. From the translational medicine perspective, the artificial implant is fabricated completely based on FDA‐approved products with elastic PDT to endow easy handling feature, thereby facilitating the clinical use, suggesting a great translational potential (Figure S22, Supporting Information). Thereby, our special design strategy, as a universal implant bioactivation technology, enables a soft‐to‐hard integration capacity for translational multi‐tissue reconstruction beyond bone‐related tissue engineering.

## 4 Experimental Section

The detailed experimental conditions and methods of synthesis, and the additional characterizations can be found in Figures S1–S22, Table S1–S3, Supporting Information. All animal experiments were performed according to protocols approved by the Shanghai Jiaotong University Animal Department (31033208).

## Conflict of Interest

The authors declare no conflict of interest.

## Supporting information

Supporting Information

Supplemental Movie 1

Supplemental Movie 2

Supplemental Movie 3

## Data Availability

Research data are not shared.
