# Supplementary material for: Bioactive Film‐Guided Soft–Hard Interface Design Technology for Multi‐Tissue Integrative Regeneration
Source: Adv Sci (Weinh). 2022 Mar 23;9(15):2105945. doi: 10.1002/advs.202105945 (PMC9130887; doi:10.1002/advs.202105945)
Supplement: Supplementary file 1 — Supporting Information [file ADVS-9-2105945-s001.pdf]

Supplementary Information

Bioactive Film-guided Soft–Hard Interface Design Technology for Multi-tissue Integrative Regeneration

Yamin Li[#], Can Chen[#], Jia Jiang[#], Shengyang Liu, Zeren Zhang, Lan Xiao, Ruixian Lian, Lili Sun, Wei Luo, Michael Tim-yun Ong, Wayne Yuk-wai Lee, Yunsu Chen, Yuan Yuan[*], Jinzhong Zhao[*], Changsheng Liu[*] & Yulin Li[*]

C. Chen, S. Y. Liu, Z. R. Zhang, R. X. Lian, L. L. Sun, W. Luo, Prof. Y. Yuan, Prof. C. S. Liu, Prof. Y. L. Li

Engineering Research Centre for Biomedical Materials of Ministry of Education

The Key Laboratory for Ultrafine Materials of Ministry of Education

School of Material Science & Engineering

Frontiers Science Center for Materiobiology and Dynamic Chemistry

East China University of Science & Technology

Shanghai 200237, China.

Email: yyuan@ecust.edu.cn; liucs@ecust.edu.cn; yulinli@ecust.edu.cn.

Dr. Y. M. Li, Prof. J. Jiang, Prof. Y. S. Chen, Prof. J. Z. Zhao

Shanghai Jiaotong University Affiliated Sixth People's Hospital

Shanghai 200233, China.

Email: jzzhao@sjtu.edu.cn.

Dr. L. Xiao

Centre for Biomedical Technologies

Queensland University of Technology

The Australia-China Centre for Tissue Engineering and Regenerative Medicine (ACCTERM)

60 Musk Avenue, Kelvin Grove, Brisbane, QLD 4059, Australia.

Prof. M. T. Ong

Department of Orthopaedics and Traumatology

Faculty of Medicine

Prince of Wales Hospital

The Chinese University of Hong Kong

Shatin, Hong Kong, China.

Prof. W. Y. Lee

Department of Orthopaedics & Traumatology

Li Ka Shing Institute of Health Sciences

Faculty of Medicine

Prince of Wales Hospital

The Chinese University of Hong Kong

Prince of Wales Hospital

Shatin, Hong Kong, China.

Experimental Section

Methods

1 Preparation and characterization of bioactive films and ligaments

1.1 Materials

Monomers racemic lactide (D, L-LA) and trimethylene carbonate (TMC) were purchased from Jinan Daigang Biomaterial Co., Ltd (China) and were used as received. Stannous caprylate Sn $(Oct)_2$ was purchased from Sigma-Aldrich (USA) and used as received. Calcium phosphate cement (CPC) was offered from Shanghai Rebone Biomaterials Co., Ltd (China). The LARS ligament graft was provided from MicroPort Scientific Corporation (China).

1.2 Synthesis of biodegradable polymer and bioactive films

Poly(lactic acid-carbonate) (PDT) was synthesized through a ring-opening polymerization method. Briefly, racemic lactide (D, L-LA) in the absence or presence of trimethylene carbonate (TMC) with a certain mole ratio were mixed under the condition of anhydrous oxygen-free at 135.0 °C for 5.5 h with $Sn(Oct)_2$ as catalyst. The product was purified by precipitation from dichloromethane with anhydrous ethanol for three times, followed by vacuum-dry to offer the pure polylactide (PDLLA) and poly(lactic acid-carbonate) (PDT).

Bioactive films were fabricated using a solvent-casting and thermo-compression technique. In brief, 6.0 g of PDT, in the presence or absence of calcium phosphate cement (CPC) powder, was dissolved in 100.0 mL of dichloromethane under ultrasonication, followed by casting into a template. The mixture underwent volatilization to get the casted pre-films, followed by thermo-compression into the PDT and PDTC films with thickness of 0.1 mm at 80.0 °C.

1.3 Fabrication of bioactive ligament graft

The PET mesh was fabricated by MicroPort Scientific Corporation. Briefly, PET meshes were fabricated from high-purity PET fibers (90.0 % crystalline) using an independent improvement Raschel warp knitting machine (ML35C Germany KarlMayer). The horizontal density (Pa) and longitudinal density (Pb) were 20.0/5.0 cm and 40.0/5.0 cm, respectively, and the machine speed was 600.0 rpm.

The bioactivated ligament graft with 0.8 mm in thickness, 10.0 cm in length and 10.0 cm in width were prepared by hot-pressing the biodegradable the PDT and PDTC films with the

LARS mesh at 150.0 °C and 10.0 MPa on a press vulcanizer (BL-6170, Bolon Instruments, China), to obtain the PDT-modified ligament (PLG) and PDTC-modified ligament (CLG). Briefly, the PDTC membrane and LARS ligament were overlapped and preheated at 80.0 °C for three minutes, exhausted for 10.0 times, then hot pressed at 10.0 MPa for five minutes, and finally cooled with water to obtain CLG graft.

1.4 Physicochemical characterization

The chemical structure of the PDT and PDTC samples was analyzed by nuclear magnetic resonance (NMR) spectroscopy with an NMR instrument (Bruker AVANCE III 600, Bruker Corporation, Switzerland). The samples were dissolved in deuterium chloroform (CD_3Cl) (Titan Technology, China) and tetramethylsilane (TMS) (Titan Technology, China) was set as the internal standard.

The molecular weight and polydispersity index of the polymers were investigated on a gel-permeation chromatographer (Waters1515, Waters, USA) using tetrahydrofuran as eluent with a flow rate of 1.0 mL min^{-1}. Polystyrene standards were used for calibration and for calculation of the weight average weight (M_w) and number average molecular weight (M_n).

The tensile testing was performed on a universal testing machine (SANS CMT 2503, MTS Industrial Systems, USA) according to GB/T 1040-2006 with strain rate of 5.0 mm/min at room temperature.

The binding force of PDT/PDTC grafts with LARS mesh was tested on a Instron distraction machine (Model 2712-004; Instron Corp) with strain rate of 5.0 mm min-1 at room temperature. The samples were prepared in following methods: the LARS mesh was cut into the size of 5.0 × 1.0 cm^2, PDT and PDTC films were cut into 1.0 × 1.0 cm^2, then two pieces of LARS mesh between which there was one piece of PDT or PDTC film were integrated in controlled thermo-pressing method. After that, the samples were tested using Instron distraction machine in accordance with the operating instructions.

Morphologies of the samples were investigated on a scanning electron microscope (SEM) (S4800, Hitachi, Japan) at an operating voltage of 15.0 kV. Energy disperse spectroscopy (EDS) (QUANTAX 400-30, Bruker AXS, Germany) analysis was performed to identify the elements present on the complex at different time points.

X-ray diffraction spectra were collected on a X-ray diffraction (XRD) (D/Max-2550VB,

Rigaku, Japan) analyzer equipped with a rotating-anode generator system using Cu Kα radiation at an operating current of 40.0 mA and a voltage of 40.0 kV. The scanning rate was 3.0 ° min^{-1}.

10.0 × 10.0 mm^2 active membrane or ligament was selected for degradation and ion release experiments. For the degradation experiment, the sample was completely immersed in 9.0 mL PBS solution at 37.0 °C. After reaching the designated time point, the samples were washed with ultrapure water, dried in vacuum, and the quality changes before and after degradation were recorded. For ion release experiment, the samples were completely immersed in 9.0 mL PBS solution at 37.0 °C and the PBS solution was updated and collected within a specified time. The calcium and phosphorus ions in the solution were detected by plasma emission spectrometer (167 nm-785 nm/725, Agilent, USA).

2 *In vitro* assays to examine the effects of materials on cell proliferation, migration, and osteogenesis

2.1 Rabbit bone marrow-derived stromal cells (BMSCs) isolation and culture

BMSCs were harvested from rabbits under sterile conditions. Briefly, rabbit femur and tibia were harvested after sacrificing, and the two ends of them were cut open, bone marrow was then flushed out of bone with 15.0 mL culture medium containing alpha minimum essential medium (α-MEM, HyClone™, GE Healthcare, UK) supplemented with 10.0 % fetal bovine serum (FBS, Gibco, USA) and 1.0 % penicillin/streptomycin (GE Healthcare, UK). The cell suspension was seeded onto a 10.0 m dish and cultured at 37.0 °C in an incubator containing 5.0 % CO_2. Cell media were half-changed every 3 days until the cells reached to 80.0-90.0 % confluence. Primary cells were passaged by treating with 3.0 mL trypsin (GE Healthcare, UK) for 1.0 min. Cells within passage 3-5 generation were used for further study.

2.2 Cell proliferation assays

Cell proliferation on PDT, PDTC films was examined by CCK-8 assay and Live-Dead staining. Rabbit BMSCs were seeded on two films in 24-well plate at a density of 2.0 × 10^4 cells/well. After 1 ,3 ,5 days of culture, the medium was changed with 0.5 mL culturing media containing 50.0 μL CCK-8 reagent (Dojindo, Japan) and then incubated at 37.0 °C for 2.0 h. Thereafter, 110.0 μL fluid from each well (of the 24-well plate) was transferred into a 96-well plate, and optical density (OD) values were measured at 450.0 nm using a microplate reader

(Labsystems Dragon Wellscan MK3, Finland). For Live-Dead staining, at each timepoint, the cell medium was removed, cells were washed with PBS and then treated with 500.0 μL Live-Dead reagent (Invitrogen, Carlsbad, CA, USA) at 37.0 °C for 15.0 min. The cells were examined using a confocal laser scanning microscope (TCS SP5, Leica, Solms, Germany).

2.3 Cell migration assay

To evaluate the effects of ligaments on the migration of BMSCs, Transwell assay was performed. Transwell inserts (8.0 μm) were purchased from Corning (NY, USA). A total of 2.0×10^4 cells was seeded onto the upper inserts and cultured with no serum α-MEM. The materials were placed in the companionate plate-well filled with α-MEM containing 3.0 % FBS. To examine the recruitment of stem cells in the material-regulated immune microenvironment, 1.0×10^5 RAW264.7 cells (SIBS, Shanghai, China) were seeded on the surface of the material, and 2.0×10^4 BMSCs were seeded onto the upper inserts. 500.0 μL DMEM (Gibco, Grand Island, NY) containing 3.0 % FBS was added to the companionate plate-well and 200.0 μL α-MEM containing 3.0 % FBS was added to the upper chamber. After 6 h (24 h) incubation, the cells stayed in the upper chamber were gently removed with cotton swabs, and cells migrated to the other side of the insert membrane were fixed with 4.0 % paraformaldehyde and then stained with 0.1% crystal violet. The number of migrated cells were imaged and counted using a light microscope (IX71SBF-2, OlympusCo, Japan)

2.4 Immunofluorescence staining

The protein levels of two representative osteogenic markers (collagen I (COL-I), bone morphogenetic protein-2 (BMP-2)) and angiogenesis marker vascular endothelial growth factor (VEGF) were examined by fluorescence staining. The cells were cultured on PDT and PDTC films with the osteogenic medium containing 10^{-7} M dexamethasone, 0.5 mM ascorbic acid, 10.0 mM β-sodium glycerate to induce osteogenic differentiation *in vitro*. After culture for 7 d the cells were fixed with 4.0 % paraformaldehyde, permeabilized with 0.1 % Triton-X for 15 min, blocked with 1.0 % Bovine serum albumin (BSA) for 30.0 min, and then incubated with primary antibodies against collagen I (COL I, Abcam, Cambridge, UK), bone morphogenetic protein-2 (BMP-2, Abcam, Cambridge, UK) and vascular endothelial growth factor (VEGF, Abcam, Cambridge, UK) overnight at 4.0 °C. Subsequently, secondary antibodies donkey-anti-mouse Alexa Fluor 488 (Abcam, Cambridge, UK) were applied to

incubate samples for 1.0 h at 25.0 ^{o}C. The cytoskeleton and nuclei were stained with phalloidin and DAPI, respectively. Images were acquired by confocal laser scanning microscope (TCS SP5, Leica, Solms, Germany).

2.5 Enzyme Linked Immunosorbent Assay (ELISA)

The secretion levels of COLI, BMP-2 and VEGF were examined with ELISA. Briefly, after culture with the films for 7 days, the cell (BMSCs) media were collected, centrifuged and stored into 2.0 mL EP tubes. The levels of Col I (ER0848, Finetest, China), BMP-2 (SEKR0038, Solarbio, China), VEGF (SEKR0009, Solarbio, China) secretion were assessed with ELISA according to the manufacture's guidelines.

2.6 Cell attachment and morphology

The attachment and morphology of rabbit BMSCs on films and ligaments were investigated by immunofluorescent staining. Cells cultured on films for 1 and 4 days or ligaments for 4 days were fixed with 4.0 % paraformaldehyde, permeabilized by 0.1 % Triton X-100 (Sigma-Aldrich, USA) for 15.0 min and blocked with 3.0 % bovine serum albumin (BSA) solution for 1 h. The cells were then stained by rhodamine-labelled phalloidin (Abcam, Cambridge, UK) for 60.0 min and DAPI (Abcam, Cambridge, UK) for 15.0 min to stain F-actin and nuclei, respectively. Images of the stained samples were obtained using a confocal laser scanning microscope (Zeiss 880, German).

2.7 Alkaline phosphatase (ALP) and alizarin red staining of BMSCs

Alkaline phosphatase (ALP) staining was used to analyze ALP expression in BMSCs. BMSCs were seeded on different films or ligaments at a density of 2.0 × 10^{4} cells/well. After 7 and 14 days of culture with osteoinductive medium, the cells were fixed with 4.0 % paraformaldehyde, washed with deionized water, and stained with ALP staining kit (Beyotime, China) for 2.0 h at 37.0 °C according to the manufacturer's protocol.

Mineralization of BMSCs was analyzed using Alizarin Red staining (ARS). After 14 and 21 days of osteogenic differentiation on films or ligaments for, the cells were fixed by 4.0 % paraformaldehyde, washed with deionized water, and stained with 1% Alizarin Red (Cyagen, China) for 30.0 min. Images of the stained samples were obtained using a Huawei camera (Honor 9, China) and light microscope (IX71SBF-2, OlympusCo, Japan).

2.8 Expression of osteogenic genes

After 7 and 14 days of culture (on LG, PLG, CLG grafts) with osteoinductive medium, total RNA was extracted from BMSCs with Trizol reagent. 1.0 μg of RNA (for each sample) was used for synthesis of complementary DNA (cDNA) using PrimeScript RT reagent kit (Takara, Tokyo, Japan) according to manufacturer's instructions. mRNA levels of osteogenic genes were measured by real-time quantitative reverse transcription-polymerase chain reaction (RT-qPCR) system (BioRad, Hercules, CA, USA). cDNA was mixed with SYBR Premix Ex Taq™ (Takara, Tokyo, Japan), forward/reverse primers and RNase free water to perform RT-qPCR. Osteogenic markers including osteocalcin (OCN), osteopontin (OPN), and osterix (Osx) were evaluated, with β-actin used as the housekeeping gene. The relative expression level for each gene (fold change) to that of LG group was calculated. All experiments were performed in triplet repeats. Primer sequences used in this study were listed in Table S3.

3 Investigating the graft-bone integration *in-vivo*

3.1 Animal model

The whole animal experiment procedure of this study was approved by the animal experiment ethics of Shanghai Jiao Tong University. A total of 27 healthy New Zealand white rabbits (around 3.0 kg) were randomly divided into three groups to receive implantation of LG, PLG, and CLG, respectively. 3.0 % pentobarbital sodium was intravenously injected at a dose of 1.0 mL kg^{-1} for anesthesia. After shaving, alcohol disinfection, and lidocaine local anesthesia, a 2.0 cm skin incision was made along the rabbit tibia nodules, followed with separation of subcutaneous tissue. A 2.5 mm bone canal was drilled horizontally from the proximal end of the tibia at both sides. The graft material (size: 2.0 × 2.0 cm^2) was rolled into a 2.5 mm-diameter solid cylinder and inserted into the bone canal to ensure that both ends of the material are exposed to the bone canal. The wound was then sterilized and closed. Penicillin was used for disinfection for 3 days after operation. The rabbits were sacrificed at 1, 3 and 6 months after surgical operation. For each rabbit, tibia at one side was harvested for the biomechanical experiment (n = 3) and tibia at another side was harvested for CT Imaging and histological examination (n = 3).

3.2 Biomechanical test

After sacrificing, the tibia was completely removed, wrapped with saline gauze, and the biomechanical experiment was performed within 4.0 h by Instron biomechanical tester. The graft was pulled out at the speed of 5.0 mm min^{-1} until the graft was completely pulled out. The maximum failure force was recorded.

3.3 Micro-computerized tomography (CT) analysis

The proximal tibias of the contralateral side were carefully dissected and fixed in formalin for 2 weeks. The specimens were placed in a sample holder and scanned at an isotropic resolution of 18.0 μm using a Micron Xray 3D Imaging System (Y. Cheetah, Germany). For each specimen, a 4.0 × 10 mm^2 cylindrical region of interest from the middle portion of the bone tunnel along its longitudinal axis was reconstructed and analyzed with the VGStudio MAX (Volume Graphics, Germany) software. The bone mineral density (BMD), bone volume/total volume (BV/TV, %), and trabecular bone pattern (TBP) factor were determined.

3.4 Histology Staining

After micro-CT tests, the samples were demineralized in 10% ethylene diamine tetraacetic acid (EDTA) for 2.0 minutes at 37.0 °C, then dehydrated and embedded in paraffin. The samples were cut into 5.0 μm slides for the following staining.

For hematoxylin-eosin (H&E), Masson trichrome and Goldner trichrome staining, the slides were dewaxed with xylene and rehydrated with graded ethanol, and then stained by hematoxylin and eosin staining reagents (Keygen, Nanjing, China), Masson trichrome (Keygen, Nanjing, China) and Masson trichrome staining reagents (Leagene, Beijing, China) according to the manufacturer’s instructions.

To detect the expression of bone morphogenetic protein-2 (BMP-2, Abcam, Cambridge, UK), vascular endothelial growth factor (VEGF, Abcam, Cambridge, UK), platelet endothelial cell adhesion molecule-1 (CD31, Abcam, Cambridge, UK), α-Smooth muscle actin (αSMA, Abcam, Cambridge, UK), Cluster of differentiation 44 (CD44, Abcam, Cambridge, UK) and runt-related transcription factor 2 (RUNX2, Abcam, Cambridge, UK), immunofluorescence (for BMP-2 and VEGF) and immunohistochemistry staining (for CD31, αSMA, CD44 and RUNX2) were performed. Briefly, after dewaxing and rehydration, the slides were incubated in 3.0 % H_2O_2 to suppress endogenous peroxidase activity. After blocking with 5.0 % BSA, the specimens were incubated with primary antibody against BMP-2 (1:200), VEGF (1:200),

CD31 (1:200), αSMA (1:200), CD44 (1:200) and RUNX2 (1:200) at 4.0 °C overnight. After washing, sections were incubated in secondary antibody or fluorescence-labelled secondary antibodies for 1.0 h at 37.0 °C. The image was observed using confocal laser scanning microscope (TCS SP5, Leica, Solms, Germany, for immunofluorescent staining) or light microscope (IX71SBF-2, OlympusCo, Japan, for immunohistochemical staining).

3.5 Foreign body response examination

A subcutaneous implantation model was created on the back of C57BL/6 mice by implanting the 10.0 × 10.0 mm^2 flat grafts (LG, PLG and CLG) subcutaneously for 1, 3, 7, 14, 28 days respectively, to investigate the foreign body response to the grafts. All the surgical procedures were performed in an aseptic manner. After the special time, the mice were sacrificed. The skin covering the grafts were harvested and fixed in paraformaldehyde. Further tissue sections (≈ 5.0 μm) were made after embedding in paraffin. Then, the sections were stained with hematoxylin and eosin (H&E) to evaluate the inflammatory reaction of the skin at different timepoints. Immunofluorescence staining was used to quantify the percentage of different phenotypes of macrophages in the fibrous layer. The staining procedures were carried out using CC-chemokine Receptor 7 (CCR7, Abcam, Cambridge, UK) (red, M1 marker) and Arginase-1 (ARG1, Abcam, Cambridge, UK) (green, M2 marker) according to the manufacturer’s instructions. The image was observed using light microscope for HE staining or confocal laser scanning microscope for immunofluorescent staining.

Statistical Analysis Results were presented as mean ± SD (standard deviation). All data were generated from at least three independent experiments and analysis was performed using SPSS analysis software (v 17.0; SPSS Inc). One-way ANOVA and the Student-Newman-Keuls post hoc test determined the level of significance in the differences between groups. A value of $p < 0.05$ was considered statistically significant.

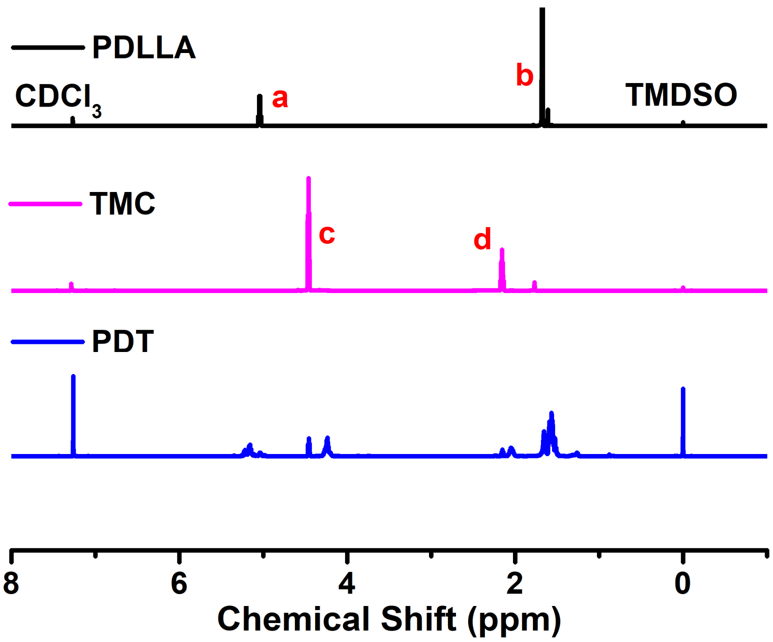

Figure S1. ^{1}H Proton Nuclear Magnetic Resonance (^{1}H NMR) of poly (D, L-lactide) (PDLLA), trimethylene carbonate (TMC) and poly (lactide-co-trimethylene carbonate) (PDT) 70:30.

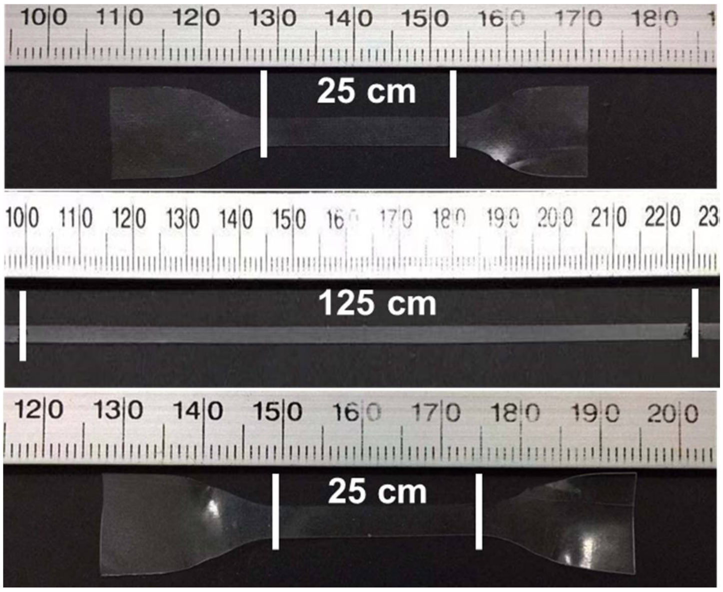

Figure S2. Shape memory properties of the hybrid film (37.0 ^{o}C, 30.0 s).

.

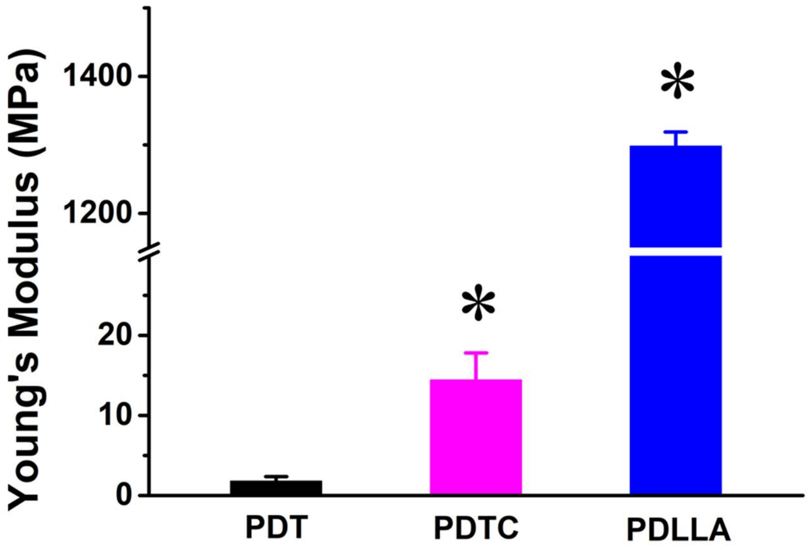

Figure S3. The Young's modulus of PDLLA, PDT and PDTC films. (*$p < 0.05$ compared to the control group, $n = 3$)

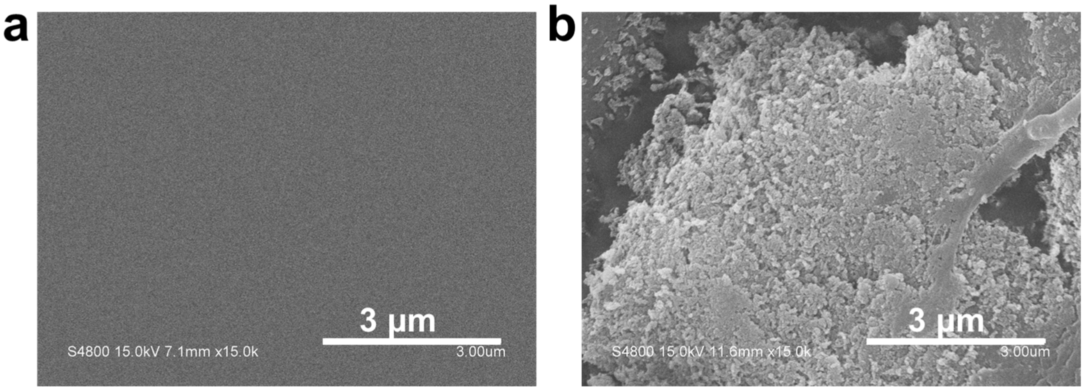

Figure S4. Scanning electron microscope (SEM) photos of the films with 96 h mineralization. a) PDT, b) PDTC.

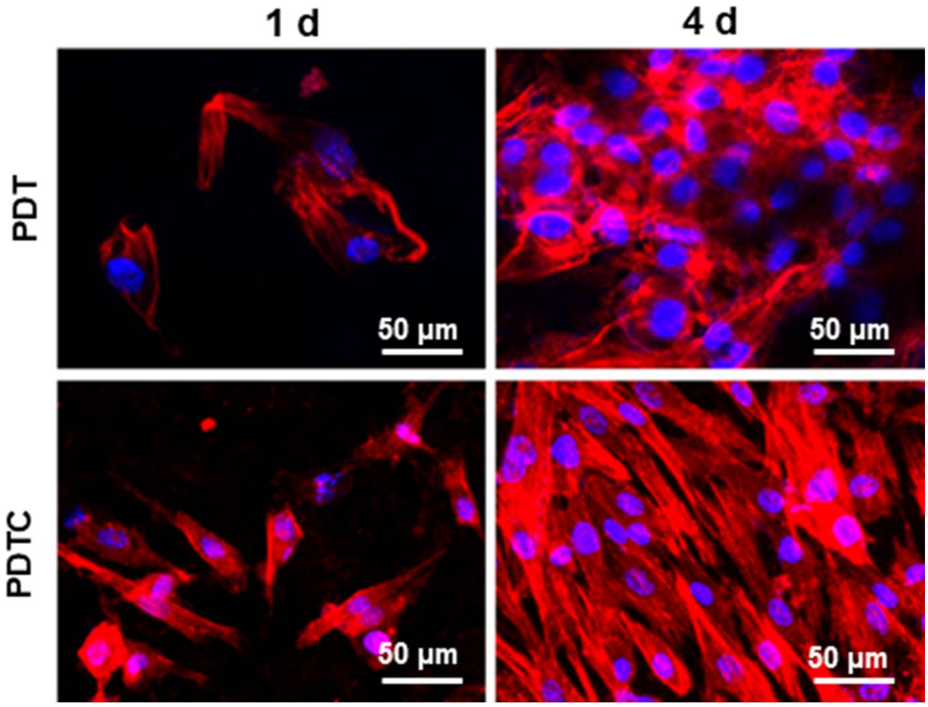

Figure S5. Cell adhesion of BMSCs cultured on the PDT and PDTC membranes for 1 and 4 days.

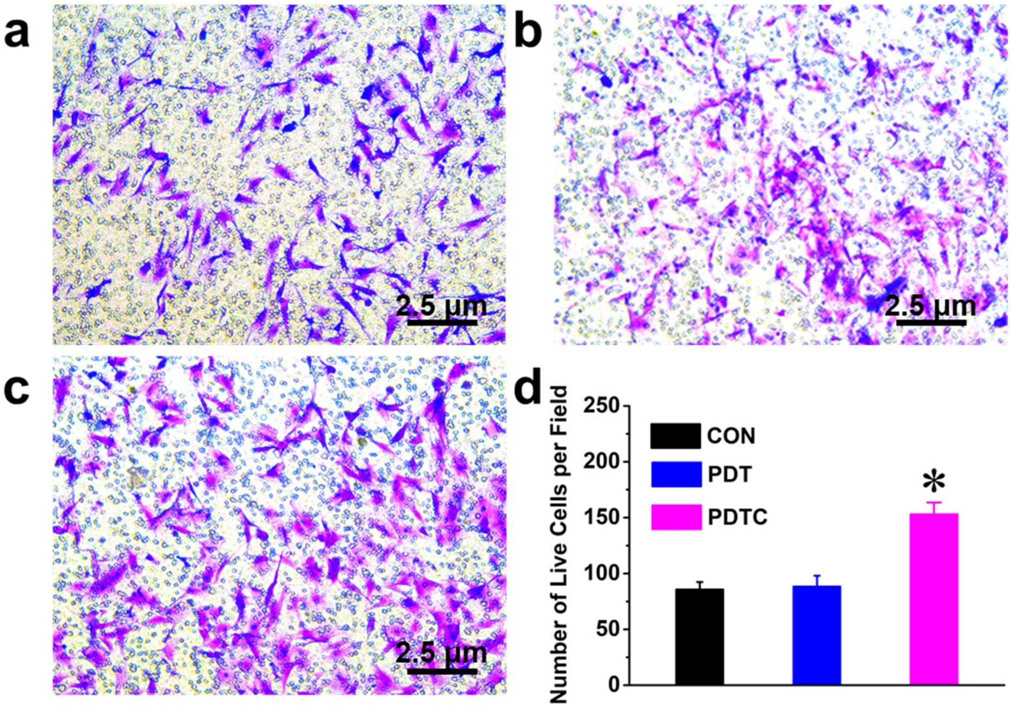

Figure S6. Cell recruitment behaviors *via* co-culturing bone marrow stromal cells (BMSCs) with macrophages for 24 h *in vitro*. a) CON, b) PDT, c) PDTC and d) the number of live cells per field. (*$p < 0.05$ compared to the control group, $n = 3$)

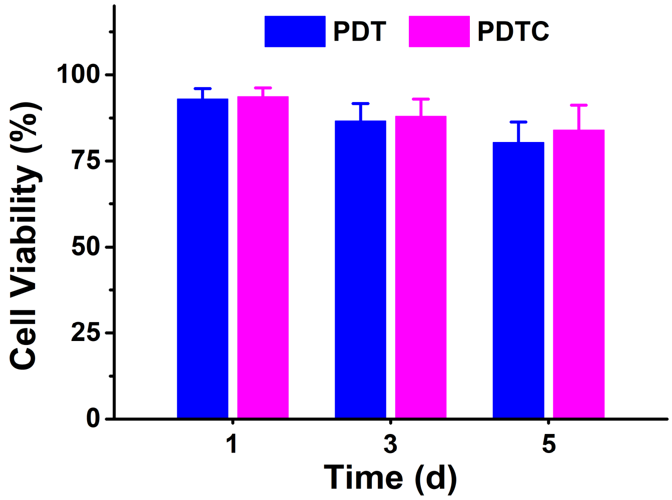

Figure S7. Cell viability of BMSCs cultured on PDT and PDTC films. (*$p < 0.05$ compared to the control group, $n = 3$)

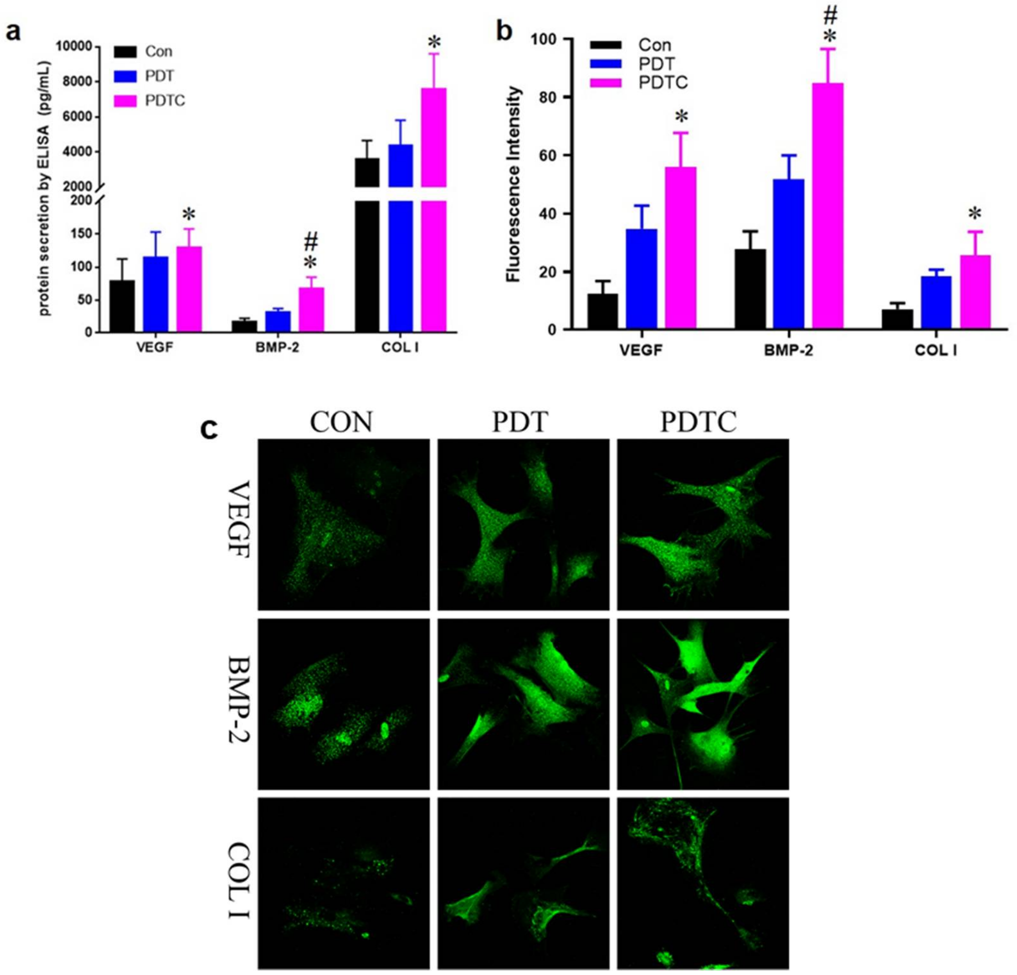

Figure S8. a) Protein secretion from BMSCs after 7 days incubation with the PDT and PDTC films. (detected by ELISA, blank cell dish served as control, $n = 3$); b) fluorescence intensity of VEGF, BMP-2, COL I in Figure S8c; c) immunofluorescence staining of VEGF, BMP-2 and Col I in the BMSCs after 7 days incubation with the PDT and PDTC films. $*p < 0.05$ compared to the control group, # $p < 0.05$ compared to the PDT group.

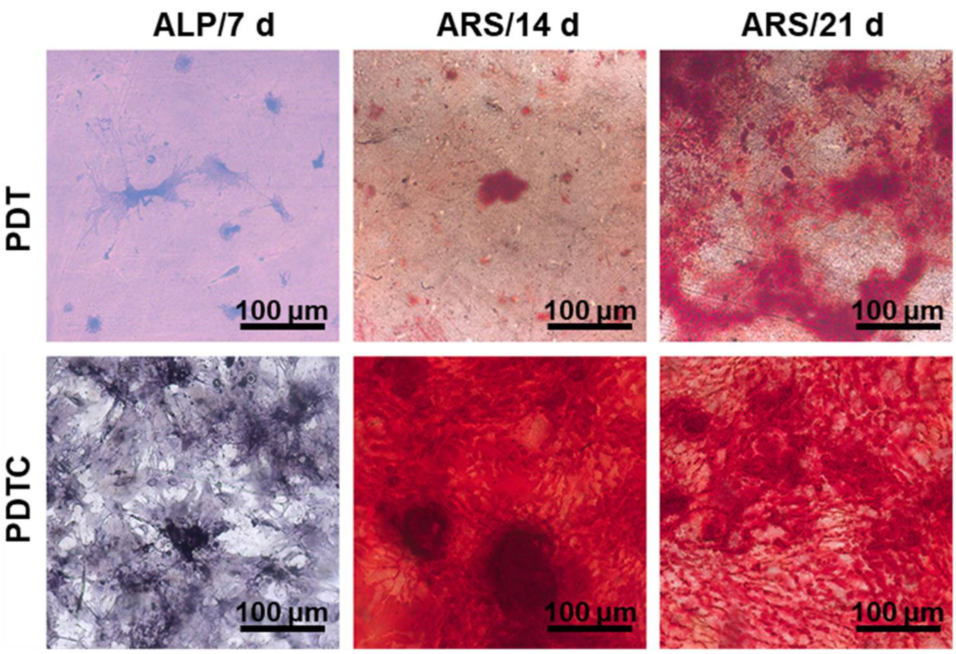

Figure S9. Alkaline phosphatase (ALP) staining and mineralization of bone marrow stromal cells (BMSCs) cultured with PDT and PDTC films. ALP analysis was performed after 7 days, and mineralization analysis was studied after day 14 and 21 d culture *via* alizarin red staining (ARS).

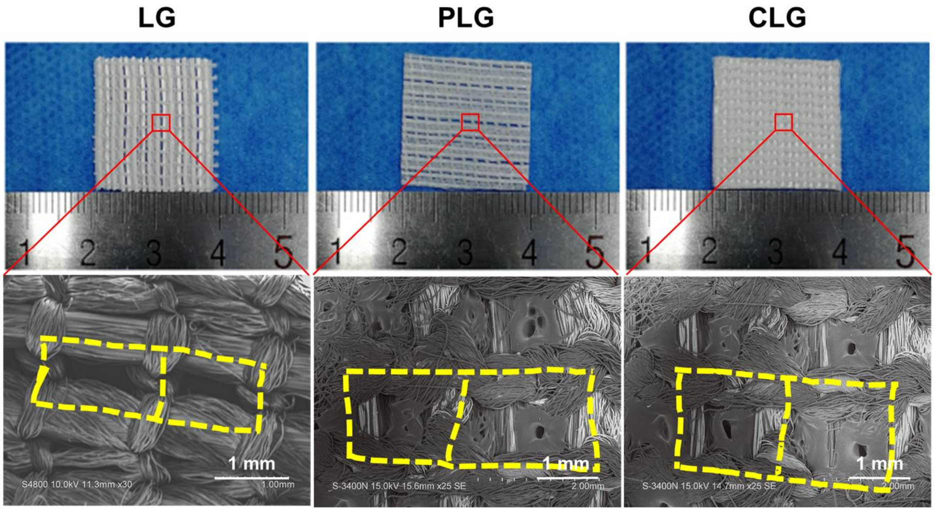

Figure S10. Schematic diagram of the ligament grafts (PLG and CLG) with preserved porous microstructure of the pure LARS ligament graft (LG).

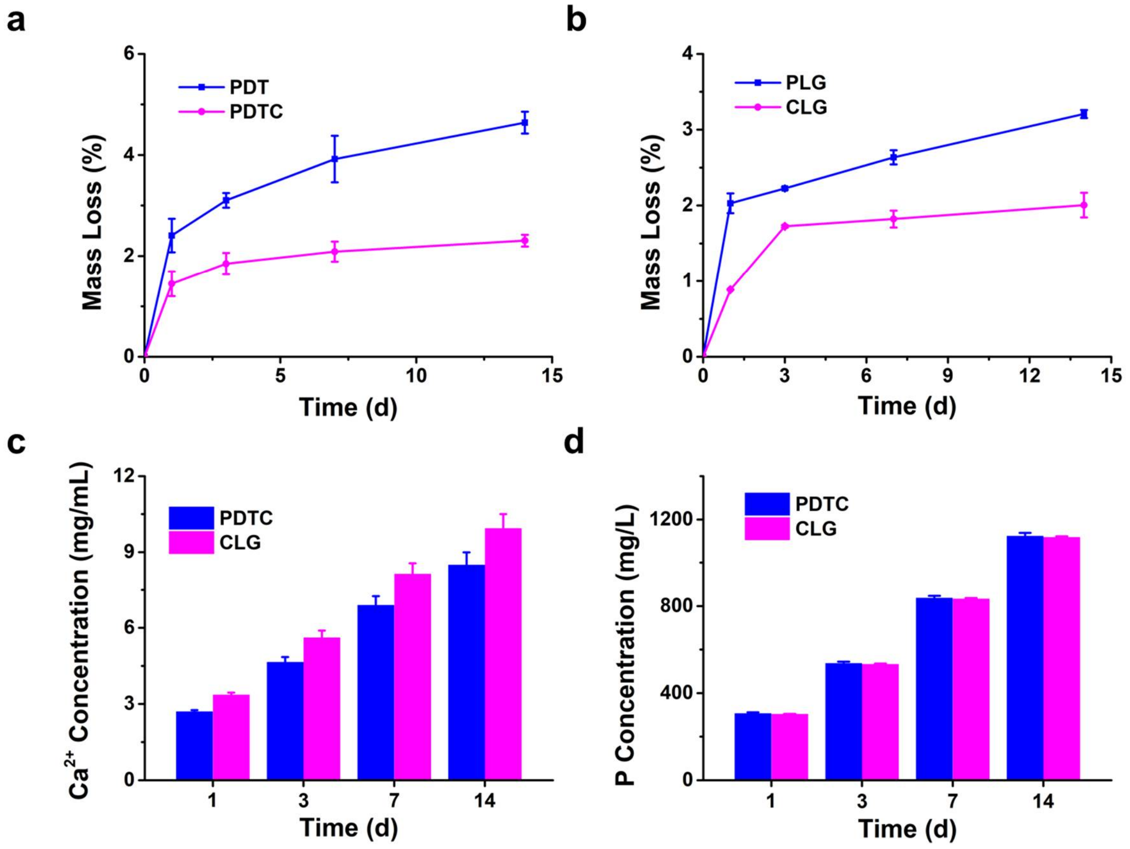

Figure S11. Degradation and ion release of bioactive membrane and graft *in vitro*. a, b) Mass loss during degradation of film and ligament. c, d) Calcium and phosphorus release curves of PDTC and CLG. (n = 3)

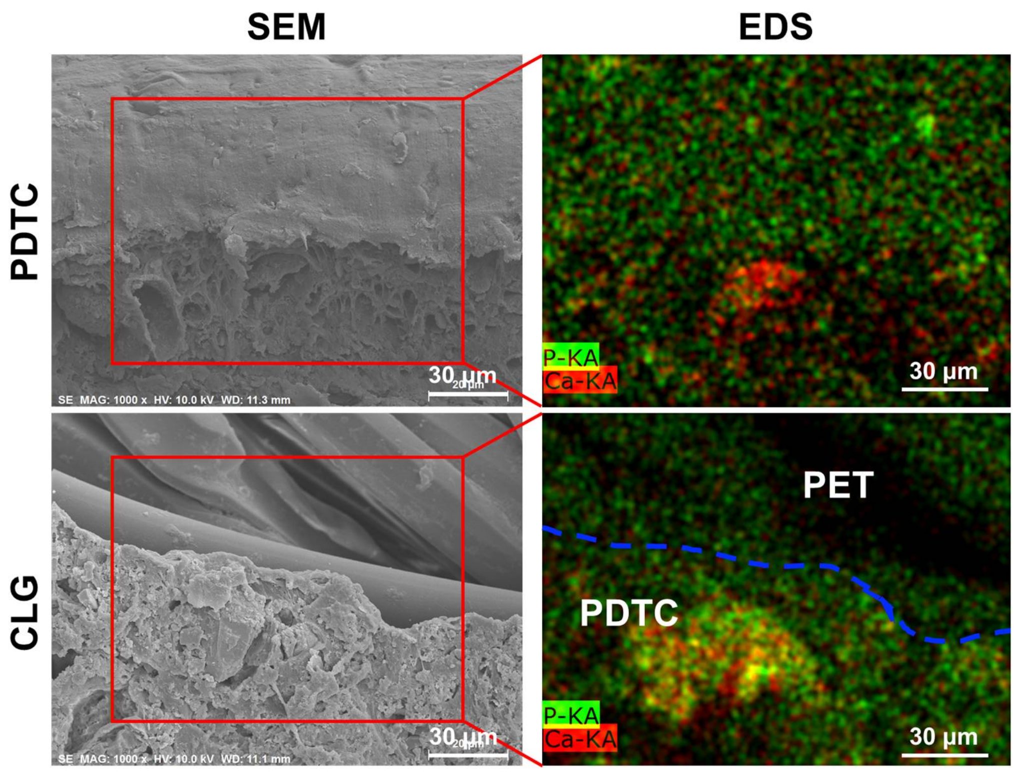

Figure S12. The energy dispersive spectrometry (EDS) of PDTC and the interface of CLG graft after 14 days of Ca and P ion release.

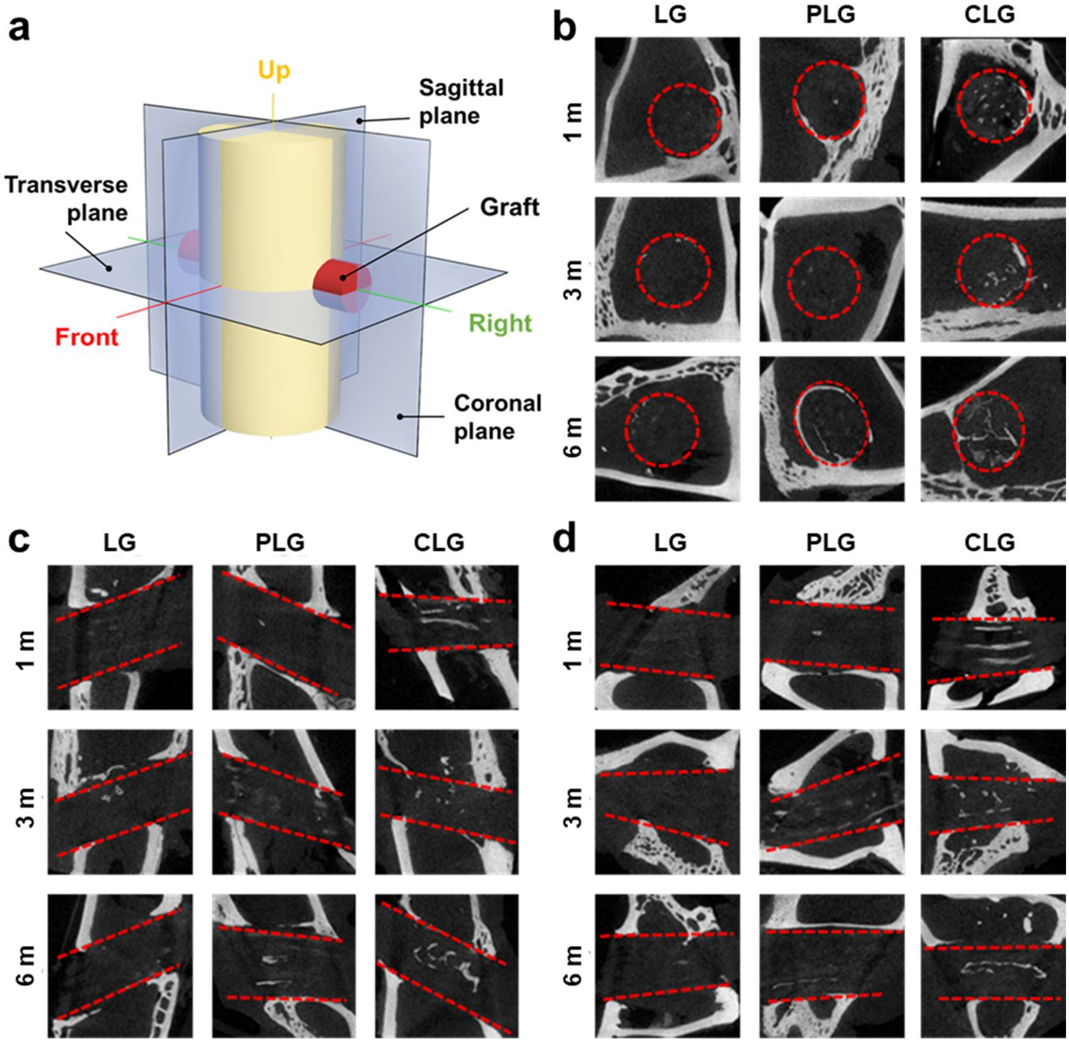

Figure S13. 3D reconstruction of micro-CT scanning showing single layer from different plates: a) schematic diagram of different plates, b) the sagittal plate, c) the coronal plate and d) the transverse plate.

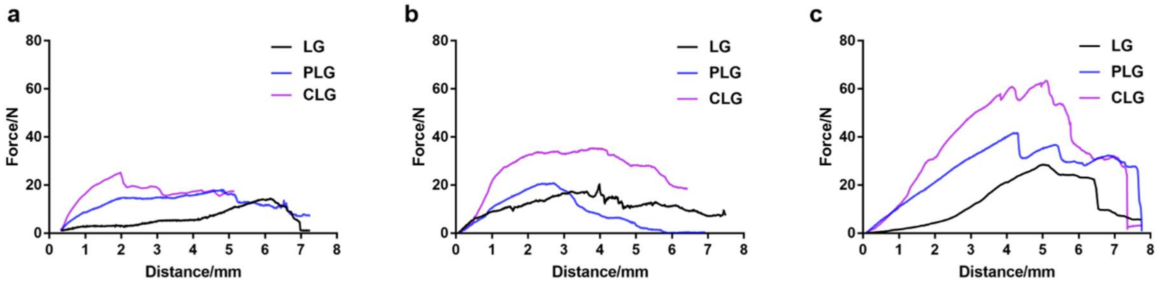

Figure S14. The osseointegration tensile force curves of LG, PLG and CLG after implantation for a) 1, b) 3 and c) 6 months.

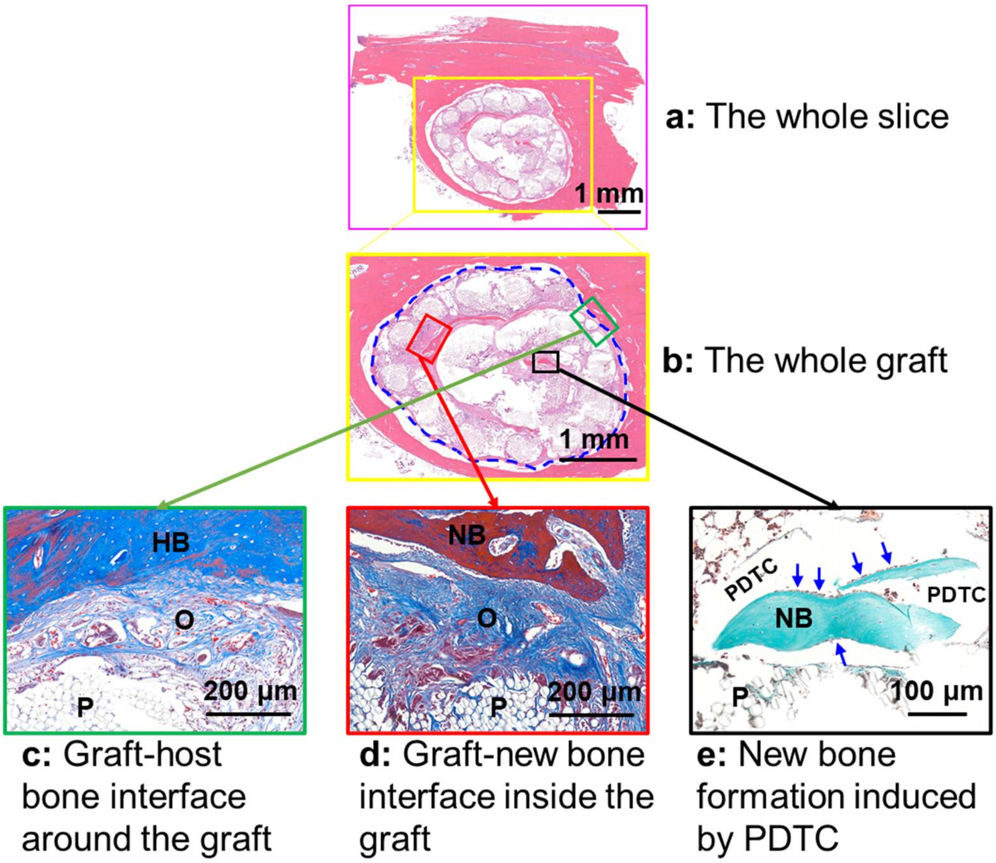

Figure S15. Illustration of the analysis processing and interest region of immunohistochemical staining (HE, Masson, Goldner) of *in vivo* samples. (P: PET; F: Fibrous tissue; B: Bone; NB: New Bone; O: Osteoid; the black arrow indicates graft-bone osteointegration.)

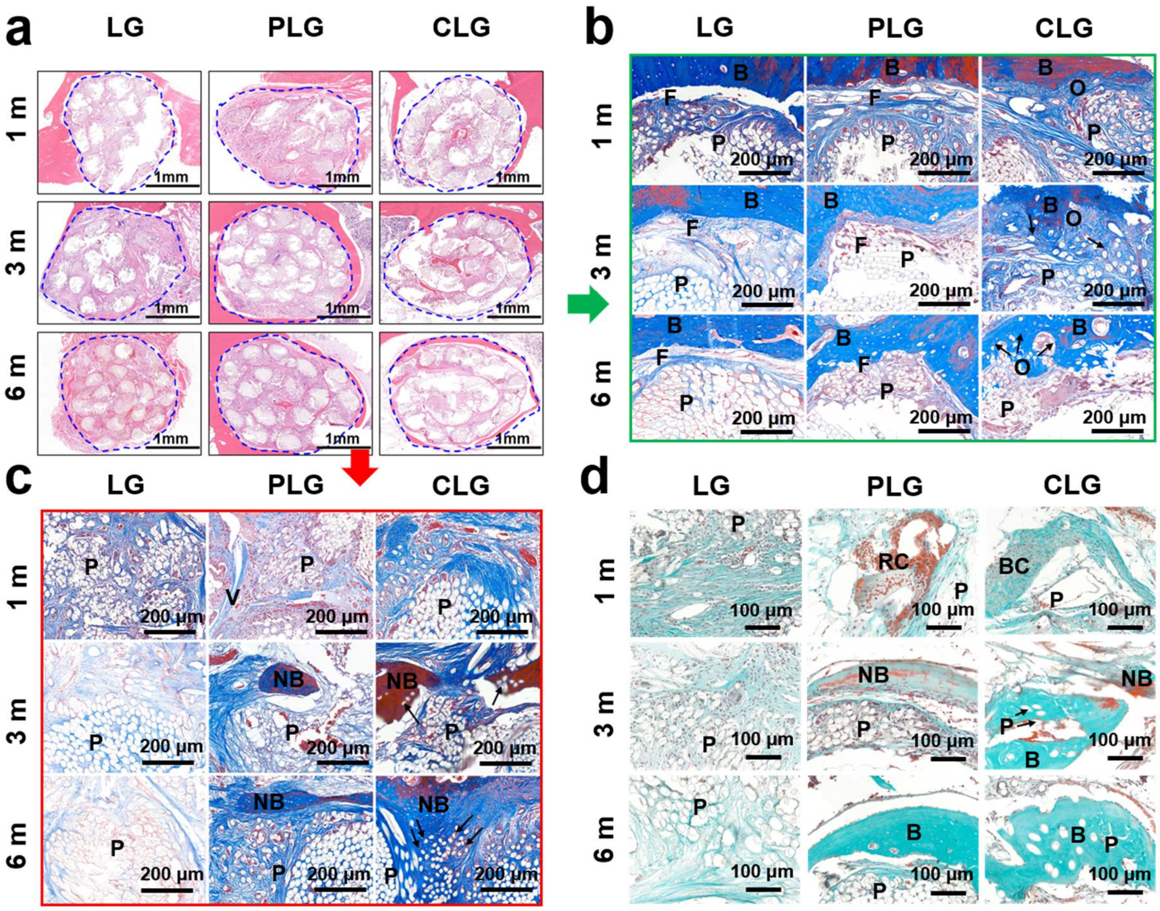

Figure S16. Histological analysis of the tissues with implanted ligament grafts *in vivo*. a-c) Represented images of HE and Masson staining of entire, boundary and interior of the ligament (PLG, CLG and LG)-bone in 1/3/6 months after implantation, the dotted blue circle indicates the implanted grafts. d) Goldner staining of the ligament (PLG, CLG and LG)-bone in 1/3/6 months after implantation. (P: PET; F: Fibrous tissue; B: Bone; NB: New Bone; V: Vessel; O: Osteoid; RC: Red Blood Cell; BC: BMSC-like Cell; the black arrow indicates graft-bone osteointegration interface.)

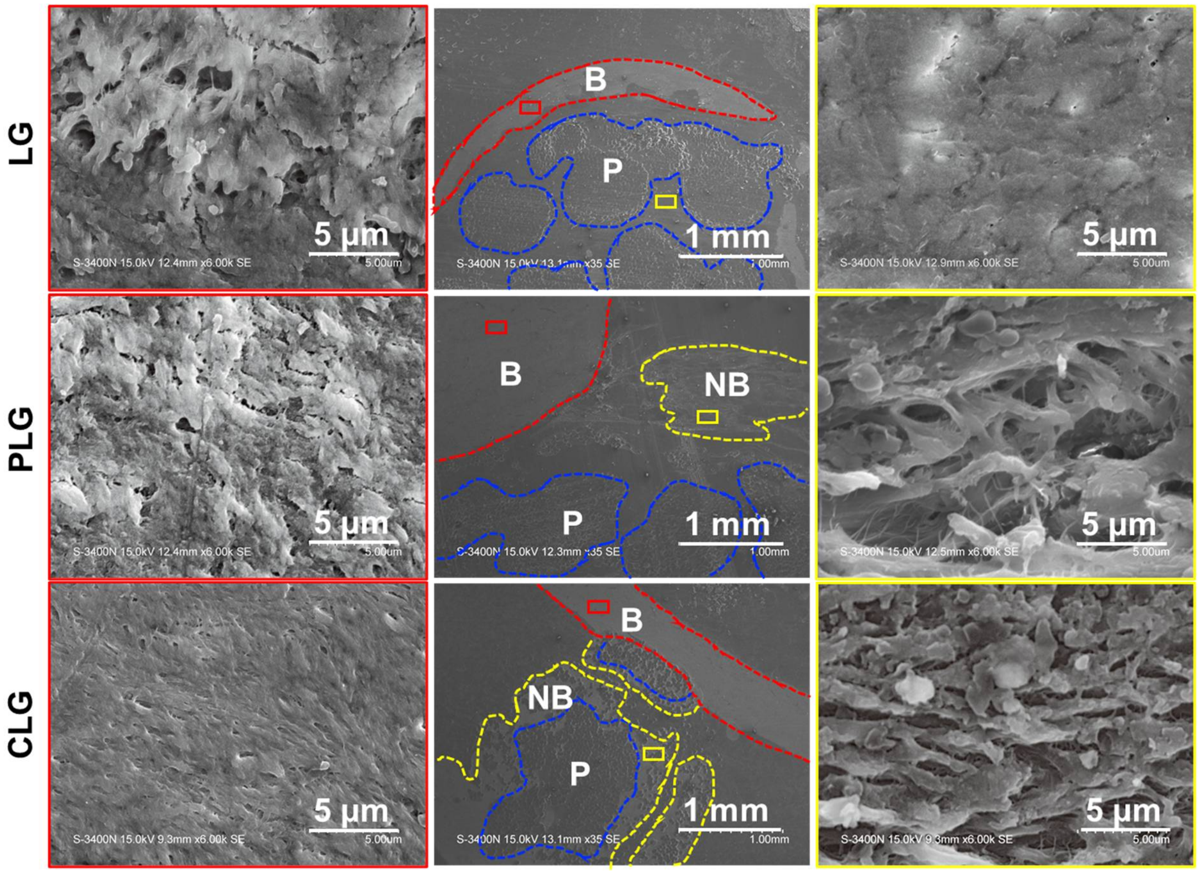

Figure S17. The SEM photos of the ligament (PLG, CLG and LG)-bone after 6 months upon implantation. (P: PET; B: Bone; NB: New Bone.)

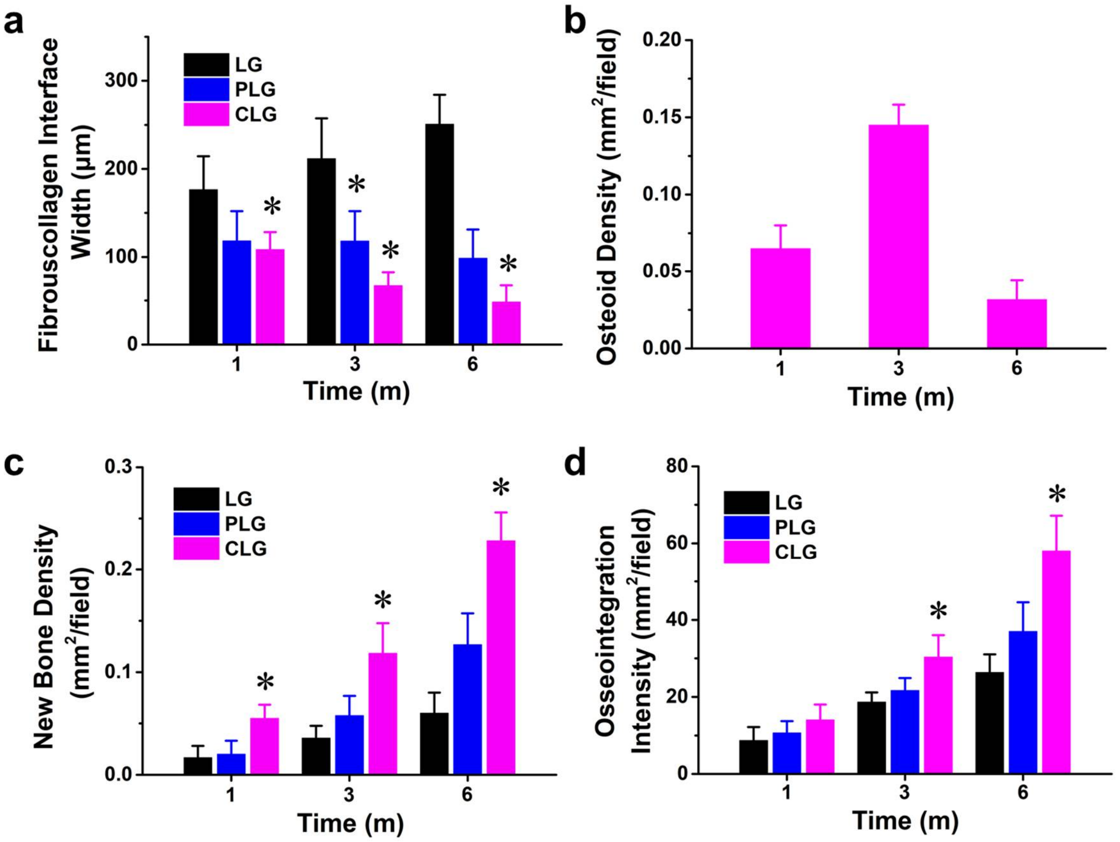

Figure S18. Statistical analysis of Figure 4 and S16. a) The fibrocollagen interface width of the ligament (PLG, CLG and LG)-bone for 1, 3 and 6 months after implantation; b) The osteoid density of CLG group for 1, 3 and 6 months after implantation; c) New bone density and d) Osseointegration intensity of the ligament (PLG, CLG and LG)-bone for 1, 3 and 6 months after implantation. (*p < 0.05 compared to the control group, n = 3)

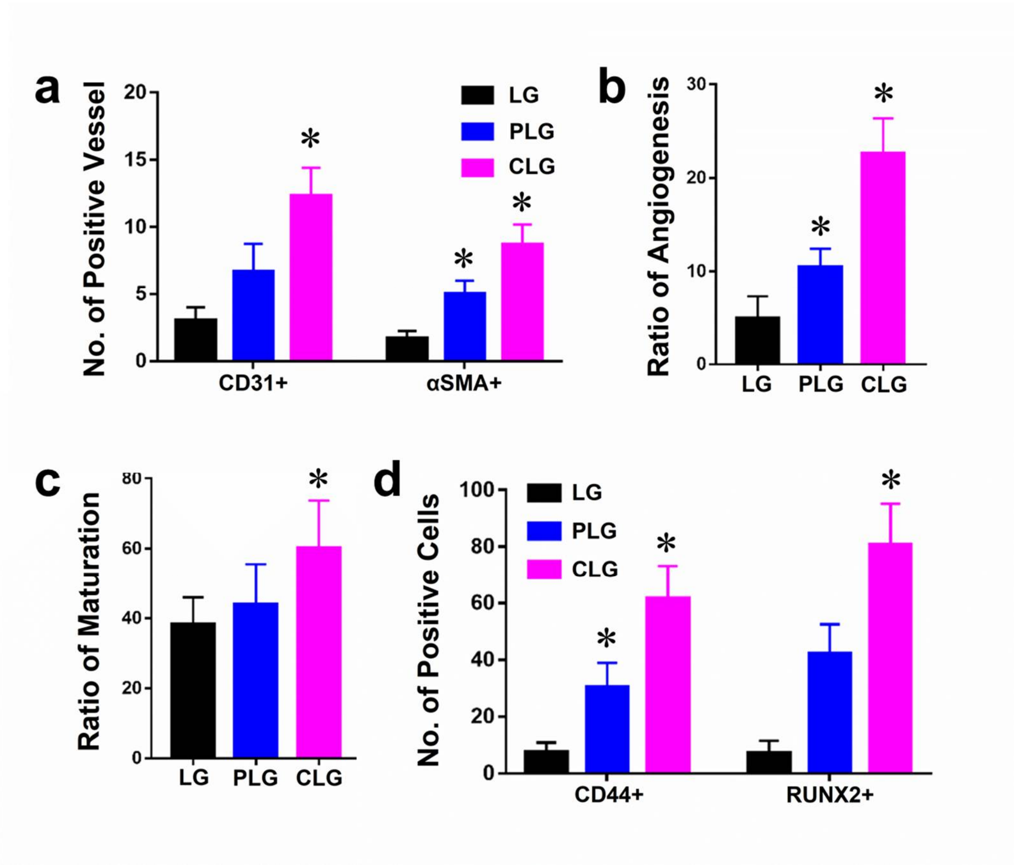

Figure S19. Statistical analysis of Figure 5. a) Number of positive vessels of CD31 and α-SMA. b) Ratio of angiogenesis ($CD31^+$ cells/total cells). c) Ratio of maturation (α-SMA/CD31). d) Number of positive cells of CD44 and RUNX2. (*$p < 0.05$ compared to the control group, $n = 3$) New bone formation is a dynamic tissue morphogenetic process that requires a series of cytokines and cells to stimulate coordinated angiogenesis and osteogenesis.[4-7] Angiogenesis indicated by CD31 and vessels mature indicated by αSMA occurs in the early stages of bone regeneration, in which VEGF plays an important role; moreover, the recruitment of endogenous BMSCs into graft site labelled by CD44 and differentiatinged osteoblasts labelled by RUNX2 are key phenomenon indicating bone regeneration, where BMP-2 is an essential protein for the later osteogenesis process[8-10]. To find out whether CLG could induce angiogenesis and enhance vessel maturation, IHC staining against CD31 and αSMA was performed. The results showed that CD31 expression was induced obviously in CLG group and moderately in the PLG group, as compared with LG group (Figures 5a and S19a). Many CD31+ tubes could be observed in CLG group, suggesting its ability to facilitate angiogenesis. The αSMA results showed that the CLG group promoted the matured bloods vessels compared to the LG and PLG group. the analysis of

angiogenesis (CD31+ cells/total cells) and ratio of maturation (a-SMA/CD31) showed that the CLG group had optimal angiogenesis and enhanced vessel maturation (Figure S19b-c). Likewise, the results of CD44 staining showed that the CLG graft significantly facilitated the recruitment and migration of CD44+ BMSCs towards the scaffolds and the RUNX2 results indicated that the CLG promoted the recruited BMSCs differentiating into RUNX2+ osteoblast (Figure 5b and S19d). In addition, upregulation of RUNX2 expression in CLG group indicated an angiogenesis-osteogenesis process was induced by CLG. To further detect the angiogenesis and osteogenesis mechanisms, we examined the expression of VEGF at month 1 (early stage) and BMP-2 at month 3 (later stage). Compared to LG ligament, CLG graft significantly upregulated VEGF expression in both bone-graft interface and the graft inside, while VEGF expression was promoted moderately in PLG group (Figure 5c), which was high around the graft, but quite low inside the graft. Accompanied with the enhanced vascularization, the expression of BMP-2 in the CLG group was drastically increased at both graft inside and graft-bone interface, as compared to the LG and PLG group (Figure 5c and S20). This phenomenon suggests that CPC may induce the accelerated production of VEGF and BMP-2, which facilitates angiogenesis and osteogenesis, respectively.

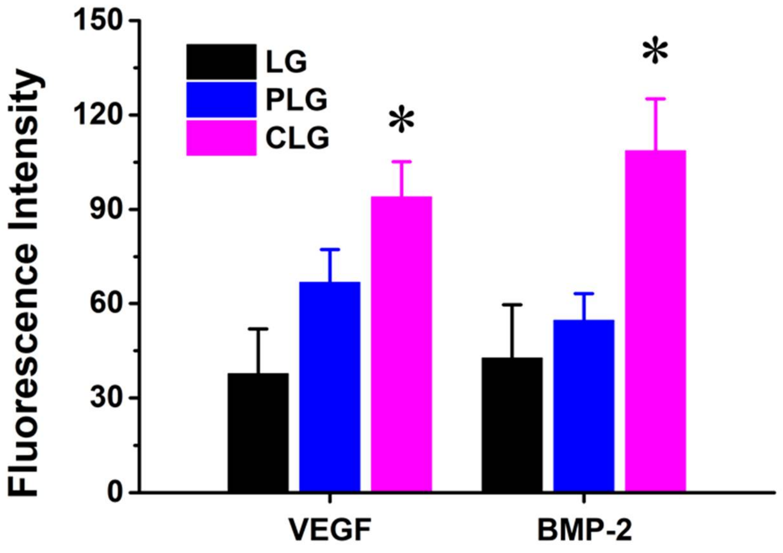

Figure S20. The fluorescence intensity of VEGF expressed after 1 m implantation, and BMP-2 after 3 m implantation at the ligament (PLG and CLG)-bone interface, with LARS ligament (LG) as control. (*$p < 0.05$ compared to the control group, $n = 3$)

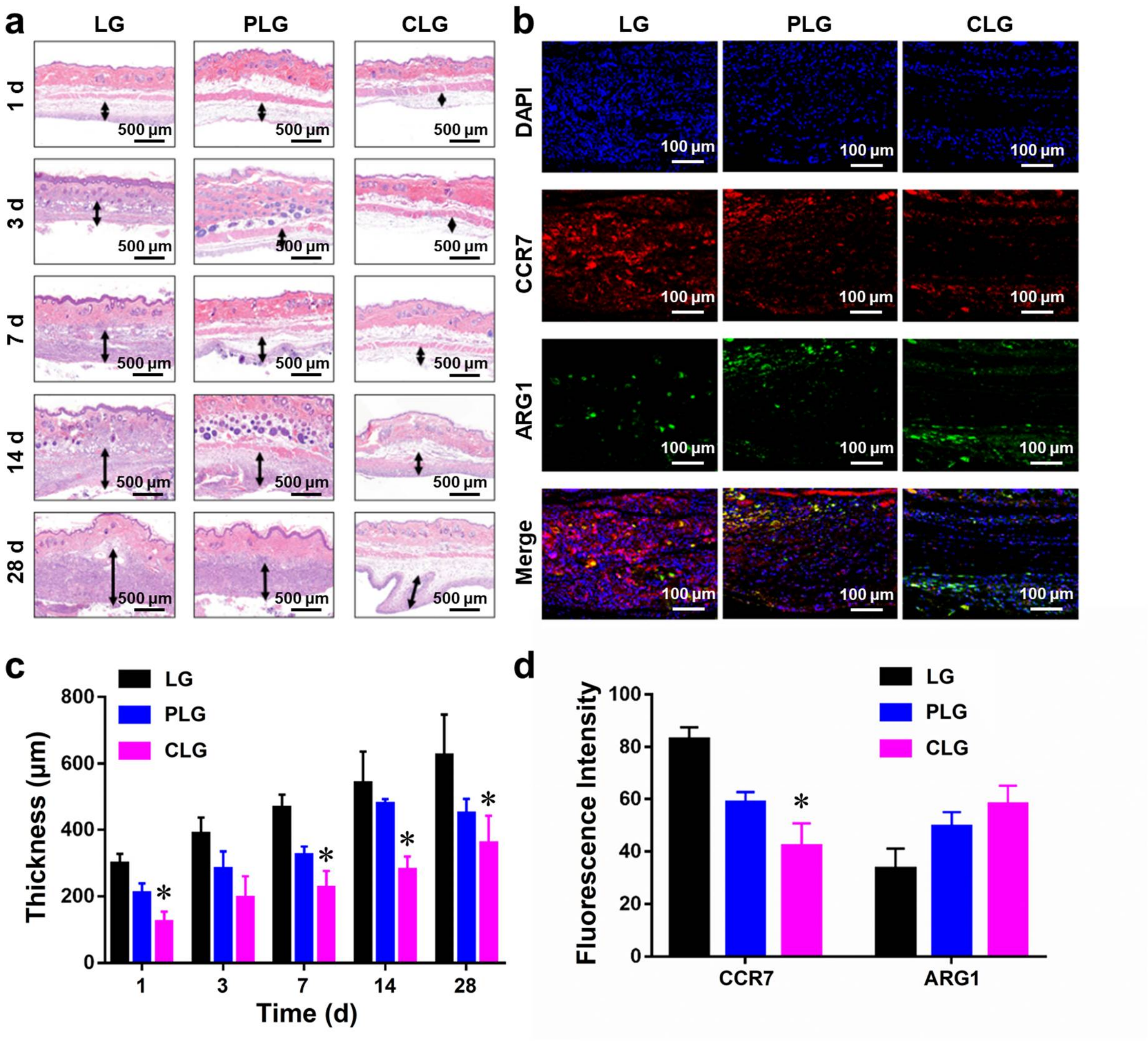

Figure S21. Foreign body response of the grafts (PLG, CLG and LG) in subcutaneous implantation model of mice. a) Representative images of HE staining. b) Immunofluorescent staining of the macrophage polarization into M1 labelled by CCR-7 and M2 labelled by ARG1 of the grafts in mice. c) Thickness of the subcutaneous inflammatory tissue. d) The fluorescence intensity of CCR7 and ARG1. (*$p < 0.05$ compared to the control group, $n = 3$)

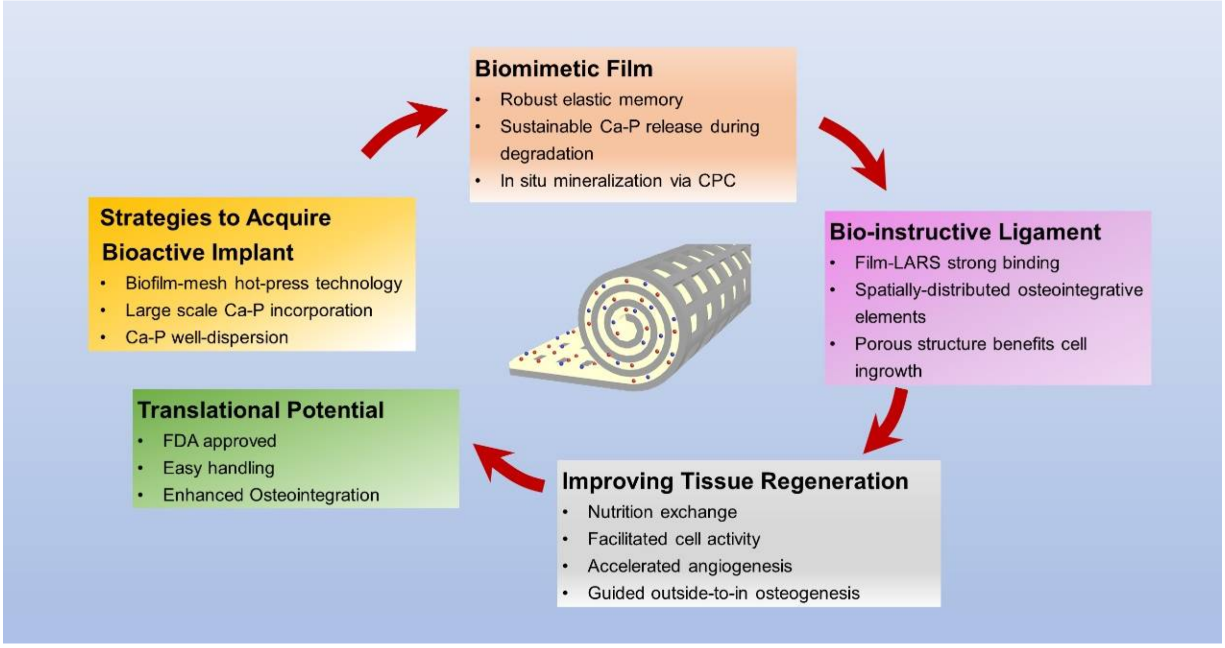

Figure S22. Schematic representation of the innovations on the bioactive ligaments with self-osteointegration capacity. 1) New Strategies to Acquire Bioactive Implant: Bioactive membrane and LARS ligament are combined with the bioactive film-mesh hot-press technology, allowing for an introduction of a large number of Ca-P bioactive compositions (CPC) to be evenly dispersed in the ligament. 2) Biomimetic Film: the bio-membrane developed in this study is a kind of degradable elastic memory membrane. During its degradation process, the Ca-P bioactive elements (CPC) with in-situ mineralization function can be released continuously. 3) Bio-instructive Ligament: Through the hot-press technology, a firm and strong combination of bio-membrane with LARS ligament could be achieved, with the good spatial distribution of CPC in the crimped active ligament, and meanwhile the native porous structure of LARS is maintained, which is conducive to the cell ingrowth inward. 4) Improving Tissue Regeneration: The porous structure of the active ligament is beneficial to the transportation of nutrients. The spatial distribution of bone-like active components and structures improve the cell activity, accelerate the angiogenesis and induce the growth of new bone from the outside to the inside. 5) Translational Potential: From a clinical point of view, the material developed in this study is synthesized from raw material approved by FDA. The bio-instructive ligaments are easy to operate in clinic and have strong osseointegration ability, which makes it an ideal ligament candidate in translational use in the future.

Table S1. The GPC result of PDT.

M_n (kDa)	M_w (kDa)	PDI
197.36	364.20	1.85

Table S2. The comparison of percentage of mechanical recovery of different grafts.

Animal	Grafts	Timepoint	Recovery Percentage
**Rabbit**	**CLG (this work)**	**6 months**	**45.7 %**
Sheep [1]	Achilles Tendon	6 months	20.0 %
Sheep [1]	Digital Flexor Tendon Autograft	6 months	20.7 %
Sheep [2]	PET Augmented Autograft	6 months	40.6 %
**Rabbit**	**CLG (this work)**	**3 months**	**26.8 %**
Rabbit [3]	Frozen Allograft	2 months	9.0 %
Rabbit [3]	Decellularized Allograft	2 months	14.4 %
**Rabbit**	**CLG (this work)**	**1 month**	**16.7 %**

Table S3. Primer sequences in RT-PCR.

Genes	Forward primer sequence	Reverse primer sequence
OCN	5’-ACCCGGGAGCAGTGTGAGC-3’	5’-GATGCGTTTGTAGGCGGTCTTC-3’
OPN	5’-TGGCTGAATTCTGAGGGACTAACT-3’	5’-ACTTTCACCGGGAGGGAGGAG-3’
Osx	5’-CCCCAGCATGTCCTACCCCAAGAT-3’	5’-GGTAGAACGCCCTGCCCCACTGC-3’
β-actin	5’-GGCCGGGACCTGACAGACTACCTC-3’	5’-GTCACGCACGATTTCCCTCTCAGC-3’

Reference:

[1] A. Weiler, G. Peters, J. Mäurer, F. N. Unterhauser, N. P. Südkamp, *Am. J. Sport. Med.* **2001**, *29*, 751-61.

[2] P. Hunt, S. U. Scheffler, F. N. Unterhauser, A. Weiler, *Arch. Orthop. Traum. Su.* **2005**, *125*, 238-48.

[3] S. K. Dong, X. Q. Huangfu, G. M. Xie, Y. Zhang, P. Shen, X. X. Li, J. Qi, J.Z. Zhao, *Am. J. Sport. Med*. **2015**, *43*, 1924-34.

[4] F. G. Khallaf, E. O. Kehinde, A. Mostafa, J. Orthop. **2016**, *13*, 69-75.

[5] G. Feng, J. Zhang, X. Feng, S. Wu, D. Huang J. Hu J, S. Zhu, D. Song, Differentiation **2016**, *92*, 195-203.

[6] A. Oryana, S. Hassanajilib, S. Sahvieh, N. Azarpirac, Life Sci. **2020**, *257*, 118038.

[7] J. Amirian, N. Linh, Y. K. Min, B. Lee, Int. J. Biol. Macromol. **2015**, *76*, 10-24.

[8] X. Wu, Y. Zhao, C. Tang, T. Yin, R. Du, J. Tian, J. Huang, H. Gregersen, G. Wang, ACS Appl. Mater. Interfaces **2016**, *8*, 7578-7589.

[9] X. Li, H. Yin, E. Luo, S. Zhu, P. Wang, Z. Zhang, G. Liao, G, J. Xu, Z. Li, J. Li, ACS Appl. Bio Mater. **2019**, *2*, 5717-5726.

[10] C. E. Kilmer, C. M. Battistoni, A. Cox, G. J. Breur, A. Panitch, J. C. Liu, ACS Biomater. Sci. Eng. **2020**, *6*, 3464-3476.
